# BCAA Catabolic Defect Alters Glucose Metabolism in Lean Mice

**DOI:** 10.3389/fphys.2019.01140

**Published:** 2019-09-04

**Authors:** Ji Wang, Yunxia Liu, Kun Lian, Xinyi Shentu, Junwei Fang, Jing Shao, Mengping Chen, Yibin Wang, Meiyi Zhou, Haipeng Sun

**Affiliations:** ^1^Department of Pathophysiology, Hongqiao International Institute of Medicine, Tongren Hospital, Key Laboratory of Cell Differentiation and Apoptosis of Chinese Ministry of Education, Shanghai Jiao Tong University School of Medicine, Shanghai, China; ^2^Department of Cardiology, Xijing Hospital, Fourth Military Medical University, Xi’an, China; ^3^School of Medicine, Shanghai Jiao Tong University, Shanghai, China; ^4^Departments of Anesthesiology, Medicine and Physiology, David Geffen School of Medicine at University of California, Los Angeles, Los Angeles, CA, United States

**Keywords:** branched-chain amino acids, glucose metabolism, catabolic defect, lean mice, liver

## Abstract

Recent studies show branched-chain amino acid (BCAA) catabolic pathway is defective in obese animals and humans, contributing to the pathogenesis of insulin resistance and diabetes. However, in the context of obesity, various processes including the dysfunctional lipid metabolism can affect insulin sensitivity and glycemic regulation. It remains unclear how BCAA catabolic defect may exert direct impacts on glucose metabolism without the disturbance of obesity. The current study characterized the glucose metabolism in lean mice in which the genetic deletion of PP2Cm leads to moderate BCAA catabolic defect. Interestingly, compared to the wildtype control, lean PP2Cm deficient mice showed enhanced insulin sensitivity and glucose tolerance, lower body weight, and the preference for carbohydrate over lipids utilization. Metabolomics profiling of plasma and tissues revealed significantly different metabolic patterns in the PP2Cm deficient mice, featured by the marked alterations in glucose metabolic processes, including gluconeogenesis/glycolysis, glycogen metabolism, and tricarboxylic acid cycle. The metabolic changes of glucose were predominantly observed in liver but not skeletal muscle or white adipose tissue. The elevated branched-chain keto acids (BCKAs) resulted from the BCAA catabolic defect may play a critical role in regulating the expression of key regulators of glucose metabolic processes and the activity of respiratory Complex II/succinate dehydrogenase in TCA cycle. Together, these results show BCAA catabolic defect significantly alters glucose metabolism in lean mice with some impacts different or even opposite from those in obese mice, highlighting the critical role of BCAA catabolism in glycemic regulation and the complex interplay between macronutrients in lean and obese animals.

## Introduction

In the past several years, insulin resistance and diabetes have been linked with disrupted branched-chain amino acids (BCAAs) homeostasis in obese animals and humans (Lynch and Adams, 2014). BCAAs, including leucine, isoleucine and valine, are essential amino acids. A number of observational studies found that elevated circulating levels of BCAAs are associated with type 2 diabetes mellitus (T2DM) and insulin resistance in humans and some rodent models (Shaham et al., 2008; Huffman et al., 2009; Tai et al., 2010; Xu et al., 2013; Lynch and Adams, 2014; Lian et al., 2015). Longitudinal and prospective studies in different cohorts have reported that increased BCAA level in blood is predictive for diabetes pathogenesis and change of plasma BCAA level is prognostic for intervention outcomes of diabetes (Wang et al., 2011; Melnik, 2012; Wang-Sattler et al., 2012; Floegel et al., 2013; Lu et al., 2013; McCormack et al., 2013). Lower BCAA level has been associated with improved insulin resistance after interventional procedures (Laferrere et al., 2011; Wang et al., 2011; Shah et al., 2012). The clear association has led to the speculation about a potential causative role of the disrupted BCAA homeostasis in T2DM (Lynch and Adams, 2014).

Branched-chain amino acid homeostasis is determined largely by their catabolic activities in tissues. The first two steps of BCAA catabolism are shared by all three BCAAs. The initial deamination step to produce branched chain keto acids (BCKAs) is catalyzed by BCAA transaminase (BCAT), which is followed by the oxidative decarboxylation to form CoA esters, a reaction catalyzed by BCKA dehydrogenase (BCKD) complex. The BCKD complex is the rate-limiting enzyme for BCAA catabolism and tightly regulated by inhibitory phosphorylation by BCKDK and activating dephosphorylation by mitochondrial phosphatase 2C (PP2Cm). Loss of PP2Cm in genetic model partially impairs BCAA catabolism, leading to the higher plasma BCAA and BCKA concentrations (Lu et al., 2009). Similarly, in obese animals and humans, BCAA catabolic genes are down-regulated and the BCAA catabolism is moderately defective, contributing to the elevated plasma BCAAs and BCKAs (She et al., 2007b, 2013; Pietiläinen et al., 2008; Herman et al., 2010; Lackey et al., 2013; Lu et al., 2013; Menni et al., 2013; Zimmerman et al., 2013).

The strong association between the elevated BCAA level and the obesity-associated T2DM indicates that disrupted BCAA homeostasis may contribute to the dysfunctional glycemic control. Indeed, recent studies show that BCAA catabolic defect contributes to the obesity-associated insulin resistance and diabetes (White et al., 2018; Zhou et al., 2019). However, in obese animals and humans, the dysregulated lipid metabolism and other processes dramatically affects insulin sensitivity and glucose metabolism. Thus, it remains challenging to distinguish the neat impacts of BCAA catabolic defect on glucose metabolism from the disturbance of obesity in obese animals. Using lean mice, the current study investigates the impacts of BCAA catabolic defect on glucose metabolic processes in a genetic mouse model in which PP2Cm is ablated to partially impair BCAA catabolism.

## Materials and Methods

### Animals

Wild type C57BL/6 and PP2Cm knockout male, age-matched mice were on the same genetic background and maintained in the same facility. PP2Cm germ-line knockout mice were generated as previously described (Lu et al., 2009). All animals (at age of 10–14 weeks) were housed at 22°C with a 12-h light, 12-h dark cycle with free access to water and standard chow. All animal procedures were carried out in accordance with the guidelines and protocols approved by the Committee for Humane Treatment of Animals at Shanghai Jiao Tong University School of Medicine or the University of California at Los Angeles Institutional Animal Care and Use Committee.

### Indirect Calorimetry Measurements

Measurements of oxygen consumption (VO_2_) and carbon dioxide production (VCO_2_) with indirect calorimetry were performed at ambient temperature using a Comprehensive Laboratory Animal Monitoring System (CLAMS, Columbus Instruments, OH, United States) according to the instructions of the manufacturer. Respiratory exchange ratio (RER) equals [volumes of CO_2_ released]/[volumes of O_2_ consumed]. Male mice were admitted to a CLAMS with free access to food and water and allowed to acclimatize in individual metabolic cages for 48 h before any measurements and the data were collected in the next 36 h.

### Glucose and Insulin Tolerance Test

Male mice were fasted for 6 h starting at 8 am. For insulin tolerance test, mice were injected intraperitoneally with insulin (0.75 U/kg body weight; Sigma, United States). For glucose tolerance test, mice were injected intraperitoneally with D-glucose (1.5 g/kg body weight; Sigma, United States). Blood glucose concentrations were measured using a portable glucometer (Johnson & Johnson, United States) through tail bleeding at the times indicated after injection.

### RNA Isolation and qRT-PCR

Total RNA was extracted from tissues or cells using the Trizol (Invitrogen, United States). Total RNA (2 μg) was reverse transcribed using random primers and MMLV (Promega, United States). Each cDNA sample was analyzed with the Applied Biosystems Prism7900HT Real-Time PCR System using Absolute SYBR Green (ABI, United States) with the following primers’ sequences:

human GYS2_F:5′CTGTAACATCCCTGGGTGGG3′,

human GYS2_R:5′GCCTCCAACTTTATTGGTCACT3′,

mouse GYS2_F:5′CCAGACAAATTCCACCTAGAGC3′,

mouse GYS2_R:5′GGGCCTGGGATACTTAAAGC3′.

human PYGL_F:5′CACTTCAGTGGCAGATGTGGTG3′,

human PYGL_R:5′GCAGTGGAAATCTGCTCTGACAG3′,

mouse PYGL_F:5′GAGAAGCGACGGCAGATCAG3′,

mouse PYGL_R:5′CTTGACCAGAGTGAAGTGCAG 3′.

### Metabolomic Analysis

The metabolomic analysis was carried out by Metabolon, Inc. (Durham, NC) using tissues from male wildtype or PP2Cm knockout mice at 14 weeks of age. After 6-h fasting starting at 8am, the animals were sacrificed by cervical dislocation. Tissue samples from white adipose tissue (epididymal fat), skeletal muscle (soleus/gastrocnemius), and liver were quickly harvested and frozen in liquid nitrogen and maintained at −80°C until processed. Samples were prepared using the automated MicroLab STAR^®^ system from Hamilton Company. To extract metabolites from tissues, extraction solution based on methanol was added to each sample in identical weight to volume ratio. The tubes containing extraction mixtures were centrifuged to precipitate proteins, and the supernatants containing metabolites were recovered for metabolomics analysis. Several types of controls were analyzed in concert with the experimental samples. The LC-MS portion of the platform was based on a Waters ACQUITY ultra-performance liquid chromatography (UPLC) and a Thermo-Finnigan LTQ mass spectrometer operated at nominal mass resolution, which consisted of an electrospray ionization (ESI) source and linear ion-trap (LIT) mass analyzer. The samples destined for analysis by GC-MS were dried under vacuum prior to being derivatized under dried nitrogen using bistrimethyl-silyltrifluoroacetamide. Derivatized samples were separated on a 5% diphenyl/95% dimethyl polysiloxane fused silica column and analyzed on a Thermo-Finnigan Trace DSQ fast-scanning single-quadrupole mass spectrometer using electron impact ionization (EI) and operated at unit mass resolving power. Raw data was extracted, peak-identified and QC processed using Metabolon’s hardware and software. Peaks were quantified using area-under-the-curve. A collection of information interpretation and visualization tools including Principal Component Analysis (PCA) and Random Forest (RF) analyses were used for data analysts. Welch’s two-sample *t*-test is used to test whether two unknown means are different from two independent populations.

### Cell Culture

HepG2 cells were cultured in Dulbecco’s modified Eagle’s medium (Hyclone, Beijing) supplemented with 10% fetal bovine serum (FBS, Sigma), penicillin (100 IU/mL) and streptomycin (100 μg/mL) in a humidified 5% CO_2_-95% air incubator at 37°C. For stimulation by BCAA (800 μM) or BCKA (400 μM), the cells were incubated in serum-free DMEM for 12 h, and then incubated in BCAA-free DMEM for 1 h before the initiation of 12 h treatments. BCAA and BCKA were diluted in BCAA-free DMEM. Custom BCAA-free DMEM was provided by Invitrogen. BCAA and BCKA chemicals were purchased from Sigma.

### Mitochondrial Assay

The isolation of mitochondria to measure oxygen consumption was performed as described elsewhere (Korge et al., 2011). Briefly, mitochondria were isolated from tissues and oxygen consumption was measured using an Ocean Optics fiber optic spectrofluorometer. Mitochondria (0.25 mg/ml) were added to the assay buffer (125 mM KCl, 10 mm HEPES-KOH, pH 7.4). The oxygen concentration in the buffer was continuously recorded via an Ocean Optics FOXY fiber optic oxygen sensor. Pyruvate, malate, and glutamate were added as free acids buffered with Tris (pH 7.4) for Complex I activity assay. Succinate was used for Complex II activity assay in presence of rotenone (1 μM). Addition of 0.2 mM ADP initiated oxygen consumption. NaCl or BCKA-Na mixture was added to the reaction system after the first pulse of ADP was consumed. Then the second pulse of ADP was added. The oxygen consumption rate (OCR) was calculated with each ADP addition. The relative rate of oxygen consumption was calculated by dividing the OCR of second pulse of ADP by the OCR of the first pulse of ADP. The presented data represented the average values of three independent experiments.

### Statistics

Unless otherwise specified, statistical analyses were performed with two-sided Student’s *t*-test or two-way ANOVA, followed by a Bonferroni *post hoc* test (tolerance tests) where appropriate using GraphPad Prism. Data were calculated as the mean ± SEM. A *p*-value of less than 0.05 was considered statistically significant.

## Results

### Physiological Characterization of the Lean Mice With BCAA Catabolic Defect

**FIGURE 1:**
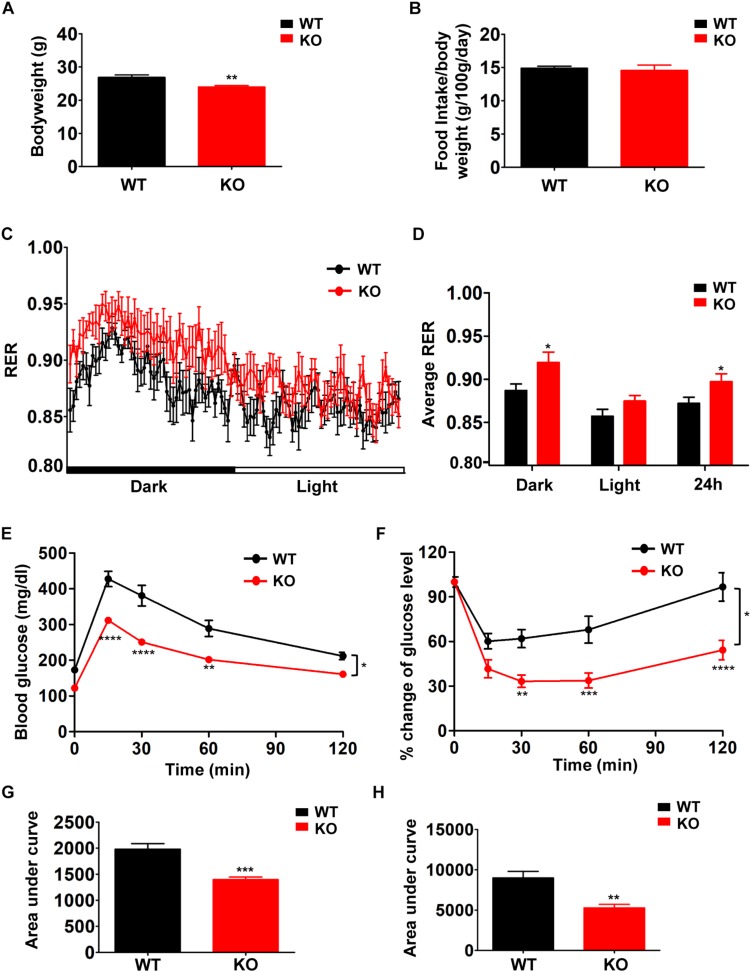
Branched-chain amino acid (BCAA) catabolic defect reduces body weight with beneficial effects on glucose metabolism in PP2Cm KO mice. Body weight (**A**, *n* = 10–11), food intake (**B**, *n* = 10–11), respiratory exchange ratios (RER) (**C**, *n* = 9/genotype), average RER during light and dark cycles (**D**, *n* = 9/genotype), glucose tolerance test **(E,G)** and insulin tolerance test **(F,H)** of WT and PP2Cm KO male mice fed a normal diet (*n* = 8 for each group). Data are represented as means ± SEM. ^∗^*p* < 0.05; ^∗∗^*p* < 0.01; ^∗∗∗^*p* < 0.001 compared to WT.

In order to examine the effects of defective BCAA catabolism on glucose metabolism in lean mice, we characterized the metabolic phenotypes of PP2Cm deficient mice in which the gene encoding PP2Cm has been genetically disrupted (Lu et al., 2009). PP2Cm is the specific BCKD phosphatase that dephosphorylates BCKDE1a subunit at Ser293 in the presence of substrates. PP2Cm deficiency partially impairs BCAA catabolism, leading to elevated plasma BCAA and BCKA concentrations. PP2Cm deficient mice showed significantly lower body weight compared with wildtype control (Figure 1A) without food intake change (Figure 1B). In indirect calorimetry, PP2Cm deficient mice showed similar energy expenditure and physical activity compared with wildtype mice (data not shown). Interestingly, the RER in PP2Cm deficient mice was significantly higher compared to that in wildtype mice, indicating PP2Cm deficient mice had an overall preference for carbohydrates as metabolic substrate (Figures 1C,D). Furthermore, glucose tolerance test and insulin tolerance test demonstrated enhanced glucose clearance and insulin sensitivity in PP2Cm deficient mice (Figures 1E–H), accompanied with an unaffected fasting plasma insulin level (data not shown). Together, these data demonstrate clear alterations of glucose metabolism in lean mice with BCAA catabolic defect.

### Metabolomics Profiling Reveals Distinguishable Metabolic Patterns of PP2Cm Deficient Mice

**FIGURE 2:**
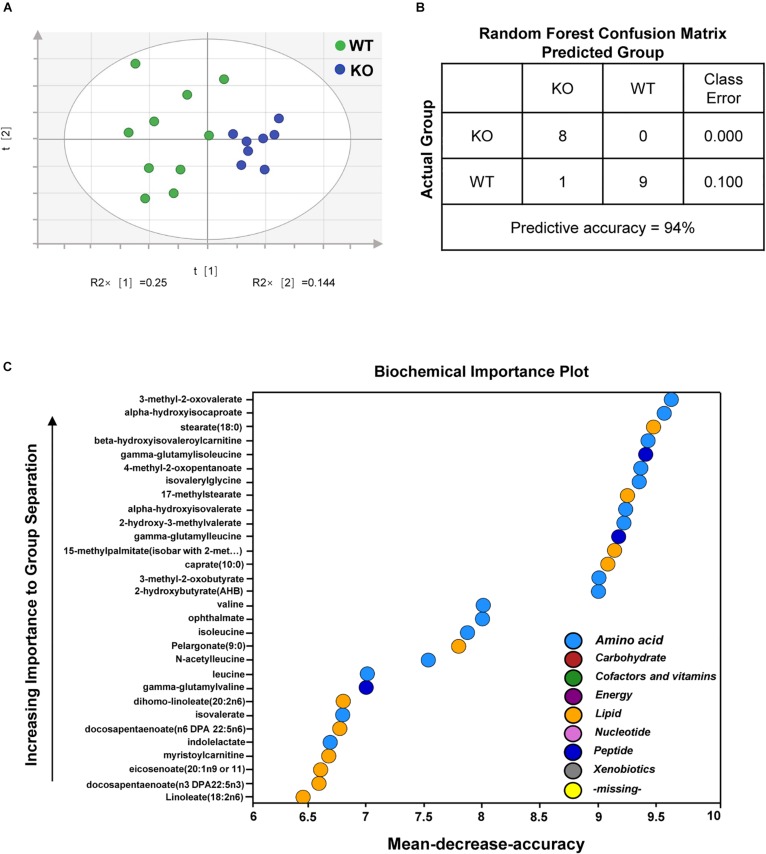
Deletion of PP2Cm resulted in significant global metabolic perturbations. **(A)** Principal component analysis (PCA) of metabolomic profiles revealed a distinct genotype-based separation for the plasma samples (WT, *n* = 10; KO, *n* = 8). **(B)** Random Forest Confusion Matrix. Random Forest classification using named metabolites detected in the plasma of wildtype (WT) and PP2Cm knockout (PP2Cm KO) mice resulted in a predictive accuracy of 94% (WT, *n* = 10; KO, *n* = 8). **(C)** List of the top 30 biochemicals that separated different genotypes based on their importance.

We next performed metabolomics profiling analyses of plasma to further characterize the biochemical changes in overall metabolism of fasted PP2Cm deficient mice. A total of 315 named biochemicals were identified and measured in mouse plasma samples. The identities and metabolic pathways of these metabolites are provided in Supplementary Table S1. Statistical comparisons revealed a large number of statistically significant differences between groups. Principal component analysis (PCA) determines if samples from different groups can be segregated based on differences in their overall metabolic signature. The PCA results illustrated a clear differentiation of PP2Cm deficient and wildtype groups (Figure 2A). Meanwhile, Random Forest (RF) analysis bins individual samples into groups based on their metabolite similarities and differences, and also defines which metabolites contribute most strongly to the group binning. RF analysis of PP2Cm deficient and wildtype mouse plasma samples resulted in a 94% predictive accuracy for assignment of individual plasma into their proper groups (Figure 2B). BCAAs and their metabolites including the BCKAs 3-methyl-2-oxovalerate, 4-methyl-2-oxopentanoate, and 3-methyl-2-oxobutyrate were among the top 30 metabolites most strongly contributing to proper group binning (Figure 2C). Together, PCA and RF analyses showed clearly distinguishable changes in the plasma metabolites between PP2Cm deficient and wild-type control mice.

### Systemic BCAA Catabolic Defect in PP2Cm Deficient Mice

**FIGURE 3:**
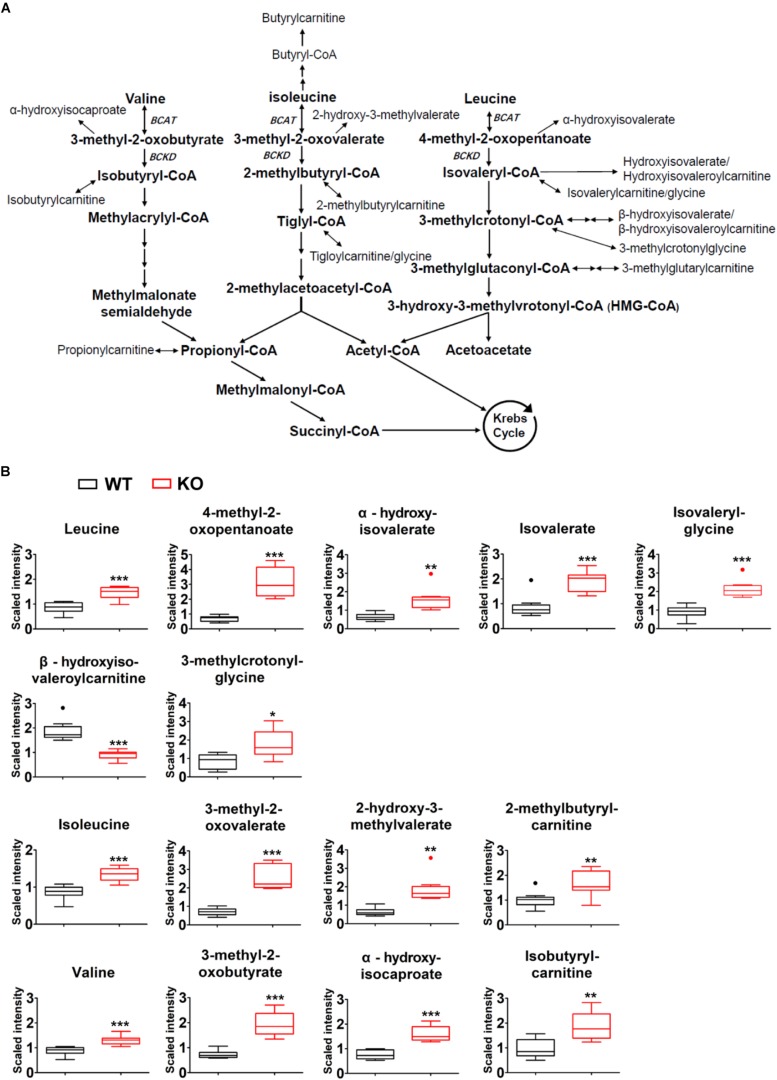
Global BCAA catabolic defect in lean PP2Cm KO mice. **(A)** Illustration of BCAA catabolic process with enzymes, intermediates, and derivatives. **(B)** Relative abundances of BCAAs and their corresponding metabolites in the plasma of wildtype (WT) and PP2Cm knockout (PP2Cm KO) mice (WT, *n* = 10; KO, *n* = 8). ^∗^*p* < 0.05; ^∗∗^*p* < 0.01; ^∗∗∗^*p* < 0.001.

Comparison of plasma global biochemical profiles for wildtype and PP2Cm deficient mice revealed several key signatures. As expected, the most dramatic effects of PP2Cm ablation was on metabolites of BCAA metabolic pathway (Figure 3A). Plasma levels of valine, leucine and isoleucine were elevated in PP2Cm deficient mice relative to wildtype mice (Figure 3B). The BCKAs, 3-methyl-2-oxobutyrate, 3-methyl-2-oxovalerate, and 4-methyl-2-oxopentanoate were also elevated in the plasma of PP2Cm deficient mice. The alpha-hydroxycarboxylic acids, 2-hydroxy-3-methylvalerate, alpha-hydroxyisocaproate, and alpha-hydroxyisovalerate, derived from reduction of the BCKAs, were all increased in the plasma of PP2Cm deficient mice (Figure 3B). Elevation of the BCAAs, BCKAs, and 2-hydroxycarboxylic acids was consistent with a decrease in BCKD activity, which was further supported by the lower abundance of beta-hydroxyisovaleroylcarnitine in the plasma of PP2Cm deficient mice (Figure 3B). Interestingly, some metabolites derived from products downstream of BCKD, including isovalerylglycine, isovalerate, 3-methylcrotonyl-glycine, 2-methylbutrylcarnitine, and isobutyrylcarnitine, were increased in the plasma of PP2Cm deficient mice (Figure 3B), suggesting complexity of BCAA catabolism from different tissues (Hutson et al., 2005). Nevertheless, genetic ablation of PP2Cm clearly causes systemic BCAA catabolic defect in mice.

### Tissue-Specific BCAA Catabolic Defect in PP2Cm Deficient Mice

To better understand how BCAA catabolic defect affects regional metabolism, we performed metabolomics analyses in liver, white adipose tissue, and skeletal muscle, the three key tissues in metabolic regulation, in fasted mice.

**FIGURE 4:**
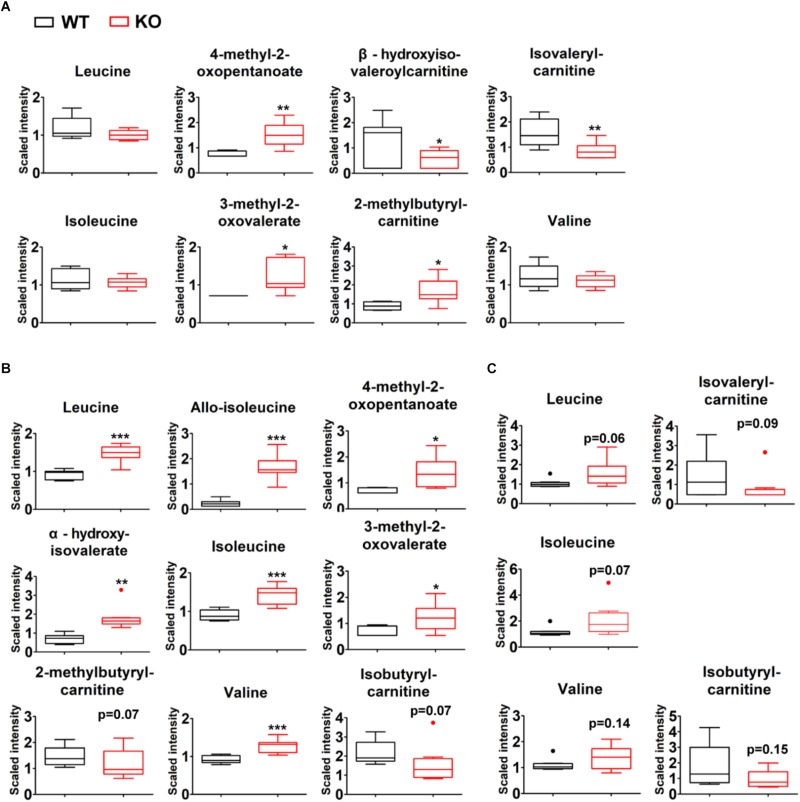
Tissue-specific BCAA catabolic defect in lean PP2Cm KO mice. Relative abundances of BCAAs and their corresponding metabolites in the liver **(A)**, skeletal muscle **(B)**, and white adipose tissue **(C)** of wildtype (WT) and PP2Cm knockout (PP2Cm KO) mice (*n* = 8 for each group). ^∗^*p* < 0.05; ^∗∗^*p* < 0.01; ^∗∗∗^*p* < 0.001.

A total of 336 compounds of known identity were identified and analyzed in liver tissues (Supplementary Table S2). Compared to wildtype counterparts, PP2Cm deficient liver possessed an accumulation of BCKAs, 4-methyl-2-oxopentanoate and 3-methyl-2-oxovalerate (Figure 4A). Lower levels of downstream BCAA catabolites including isovalerylcarnitine and beta-hydroxyisovaleroylcarnitine were observed in PP2Cm deficient liver (Figure 4A). These changes reflected the impaired BCKD activity due to the ablation of PP2Cm. It was unexpected that the BCAA levels were not higher while 2-methylbutyrylcarnitine was elevated in the PP2Cm deficient liver (Figure 4A).

A total of 317 chemicals of known identity were detected in skeletal muscle samples (Supplementary Table S3). BCAA and BCAA catabolic products formed upstream of BCKD, including 3-methyl-2-oxovalerate, 4-methyl-2-oxopentanoate, alpha-hydroxyisovalerate, and allo-isoleucine, were elevated in the skeletal muscle from PP2Cm deficient mice (Figure 4B), consistent with defective BCKD activity. In addition to these changes, metabolites formed downstream of BCKD (isobutyrylcarnitine and 2-methylbutyrylcarnitine) were found with trends of lower levels in the skeletal muscles from PP2Cm deficient animals compared to wildtype counterparts (Figure 4B). These changes demonstrated the impaired BCKD activity and the defect in BCAA catabolism in PP2Cm deficient skeletal muscle.

A total of 180 compounds of known identity were detected in adipose tissue samples (Supplementary Table S4). PP2Cm deficient adipose tissue possessed trends of elevated BCAAs compared to wildtype counterparts (Figure 4C). Furthermore, the BCKD downstream catabolites isovalerylcarnitine and isobutyrylcarnitine showed trends of decrease in PP2Cm deficient adipose tissue compared to that of wildtype mice (Figure 4C).

Together with the plasma profiles, these data suggest that PP2Cm ablation leads to the whole-body BCAA catabolic defect, accompanied with tissue-specific patterns.

### Impacts of BCAA Catabolic Defect on Glycogen Metabolism

Besides the defective BCAA catabolism in PP2Cm deficient mice, significant alterations were detected by metabolomics analyses in glucose metabolic processes including glycogenesis/glycogenolysis, glycolysis/gluconeogenesis, and TCA cycle in different tissues of fasted mice.

**FIGURE 5:**
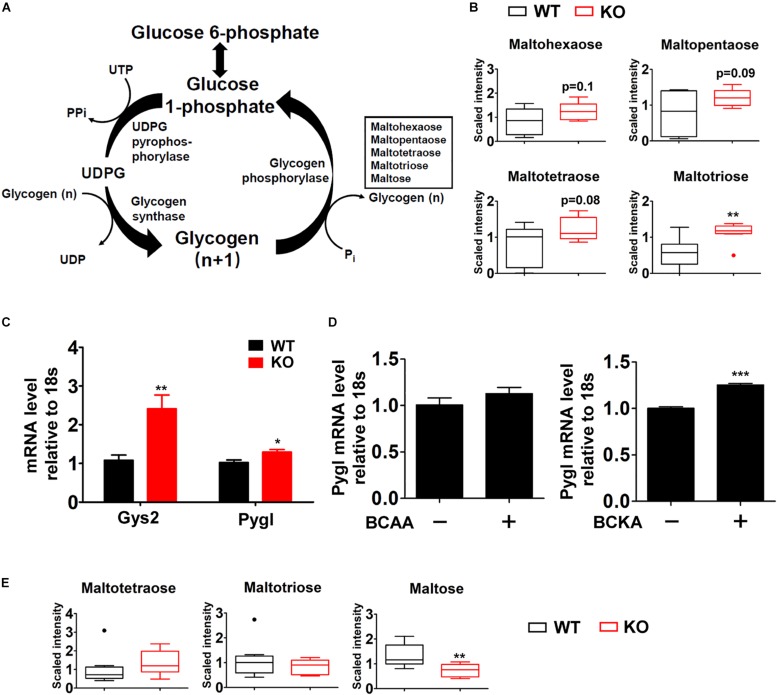
Box plots of various metabolites in glycogen catabolic pathway altered in the PP2Cm KO mice. **(A)** Illustration of glycogen metabolic pathway with enzymes and intermediates. **(B)** Significantly altered metabolites in liver are shown as box plots. ^∗^*P* < 0.05; **(C)** Real-time RT-PCR result of specific genes using mRNA from liver of wildtype (WT) and PP2Cm knockout (PP2Cm KO) mice (*n* ≥ 9 for each group). Data are represented as means ± SEM. ^∗^*p* < 0.05; ^∗∗^*p* < 0.01. **(D)** mRNA levels of PYGL in BCAA and BCKA treated HepG2 cells (*n* = 3 for each group). Data are represented as means ± SEM. ^∗^*p* < 0.05; ^∗∗^*p* < 0.01; ^∗∗∗^*p* < 0.001. **(E)** Significantly altered metabolite in skeletal muscle (*n* = 8 for each group). ^∗^*p* < 0.05; ^∗∗^*p* < 0.01.

One major change was observed in glycogen metabolism in liver of fasted P2Cm deficient mice. Glycogen is the storage form of glucose in liver and skeletal muscle. Glycogen synthesis (glycogenesis) is important for blood glucose disposal in the fed state. During short-term fasting periods, the liver releases glucose mainly through glycogenolysis, contributing to the maintenance of blood glucose (Figure 5A). PP2Cm deficient liver possessed significantly higher level of the glycogen metabolite maltotriose and trends of elevated maltohexaose, maltopentaose, and maltotetraose compared to wildtype counterparts (Figure 5B). Meanwhile, PP2Cm deficient liver showed higher mRNA expression of glycogen phosphorylase (PYGL) and glycogen synthase (GYS2), the controllers of glycogenolysis and glycogenesis in liver, respectively (Figure 5C). Interestingly, BCKAs, but not BCAAs, induced PYGL expression in HepG2 cells (Figure 5D). The expression of GYS2 in HepG2 was low and the impacts of BCAAs and BCKAs on it remained unclear. These results indicate that BCAA catabolism defect alters glycogen metabolism in liver.

In contrast to liver tissue, glycogen metabolites maltose was markedly decreased in PP2Cm deficient skeletal muscle while maltotriose and maltotetraose showed no significant changes (Figure 5E), indicating different effects of BCAA catabolic defect on glycogen metabolism in liver and skeletal muscle.

### Impacts of BCAA Catabolic Defect on Glycolysis/Gluconeogenesis

**FIGURE 6:**
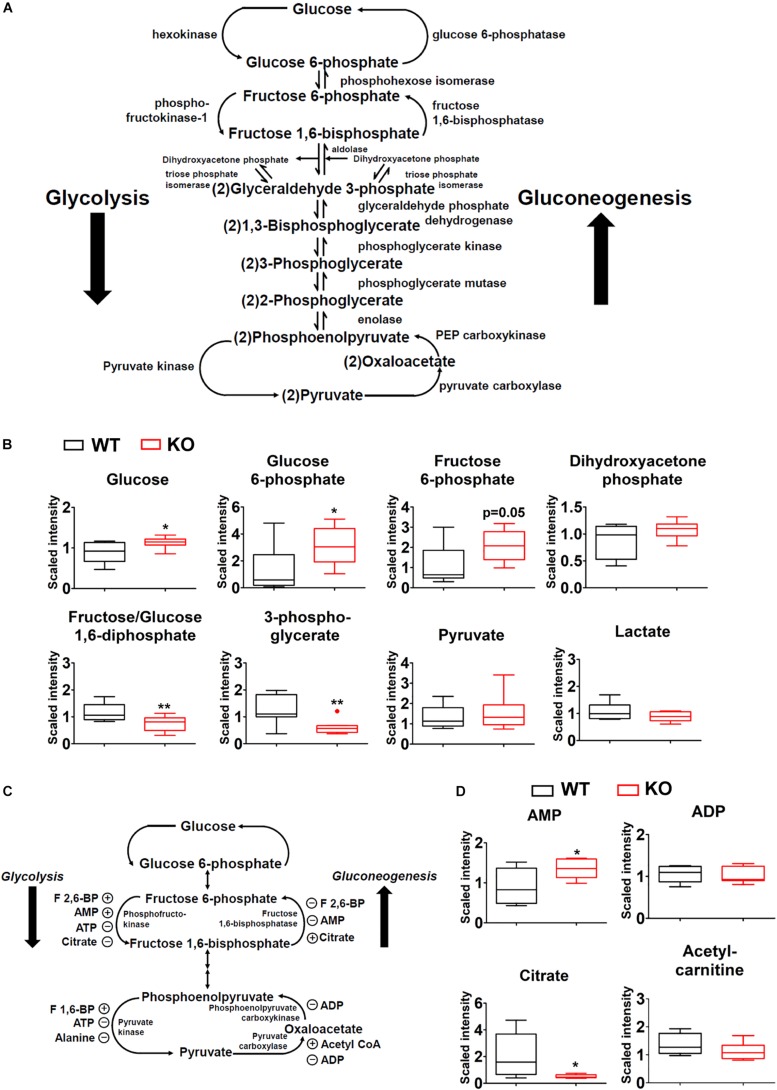
Impacts of BCAA catabolic defect on glycolysis/gluconeogenesis in liver. **(A)** Illustration of glycolysis/gluconeogenesis with enzymes and intermediates. **(B)** Metabolomics analysis results showing the relative levels of glycolysis/gluconeogenesis intermediates in PP2Cm KO liver (*n* = 8 for each group). **(C)** Illustration of glycolysis/gluconeogenesis with enzymes and allosteric modulators. **(D)** Metabolomics analysis results showing the relative levels of allosteric modulators of glycolysis/gluconeogenesis in PP2Cm KO liver (*n* = 8 for each group). ^∗^*p* < 0.05, ^∗∗^*p* < 0.01.

Glycolysis and gluconeogenesis are two central processes of glucose metabolism in reverse direction, sharing enzymes and metabolic intermediates (Figure 6A). The abundances of glucose, glucose-6-phosphate, and likely fructose-6-phosphate (upstream metabolites of glycolysis) were increased in PP2Cm deficient liver while fructose-1,6-biphosphate and 3-phosphoglycerate (downstream metabolites of glycolysis) showed lower levels (Figure 6B). No significant differences were detected for pyruvate, lactate, and dihydroxyacetone phosphate (Figure 6B).

Allosteric modulators of glycolysis/gluconeogenesis include acetyl-CoA, AMP, ADP, citrate, F-2,6-BP (Figure 6C). The metabolomics analyses detected lower citrate, unchanged acetyl-carnitine, unchanged ADP, and higher AMP in the PP2Cm deficient liver, which likely enhanced glycolysis but not gluconeogenesis (Figure 6D).

In contrast to liver, no significant differences in glycolic intermediates were observed between PP2Cm deficient and wildtype adipose tissues or skeletal muscle, respectively (Supplementary Figure S1).

### Impacts of BCAA Catabolic Defect on TCA Cycle

**FIGURE 7:**
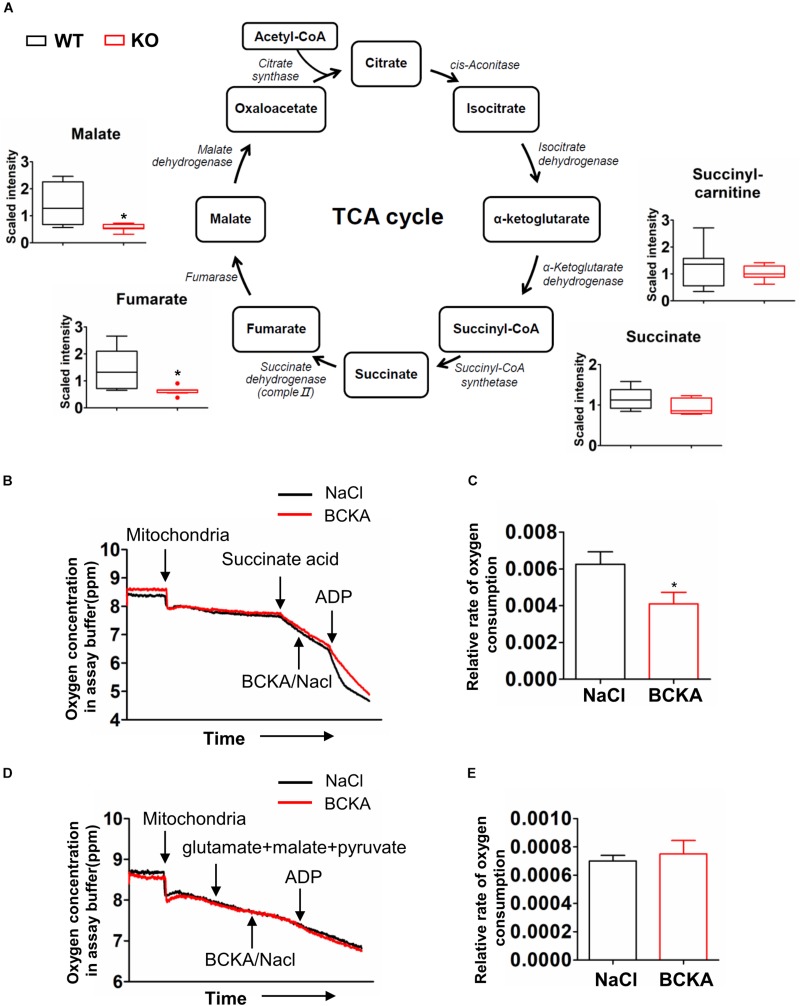
Impacts of BCAA catabolic defect on TCA cycle. **(A)** Metabolomics analysis results showing the relative levels of metabolites mapped onto the TCA cycles in liver of wildtype (WT) and PP2Cm knockout (PP2Cm KO) mice (*n* = 8 for each group). **(B–E)** Oxygen consumption in mitochondria isolated from wildtype liver in absence or presence of BCKA-Na (500 μM each of KIC, KIV, KMV mixed) using Complex II **(B,C)** or Complex I **(D,E)** substrates, respectively. NaCl (1.5 mM) was used as control. *Y*-axis: oxygen concentration (ppm) in assay buffer. The assay was completed in ∼12 min. **(C,E)** Relative oxygen consumption rate in the absence or presence of BCKAs calculated based on results in panels (**B** or **D**) (*n* = 3 in each group; Data are represented as means ± SEM. ^∗^*p* < 0.05 vs. control), respectively.

Glycolysis generates pyruvate, which subsequently is oxidized in the tricarboxylic acid (TCA) cycle. The metabolomics analyses detected lower abundances of multiple TCA cycle intermediates including fumarate, malate, and citrate (Figure 6D) in PP2Cm deficient liver compared to wildtype counterpart (Figure 7A).

It has been shown that BCKAs inhibited mitochondrial respiration in cardiac mitochondria (Sun et al., 2016). The enzyme that catalyzes the conversion from succinate to fumarate in TCA cycle is succinate dehydrogenase, which is also the respiration Complex II in the electron transfer chain in mitochondria (Figure 7A). The lower abundances of fumarate and malate but not succinate indicated the inhibition of succinate dehydrogenase/Complex II. BCKAs was elevated in the PP2Cm deficient liver (Figure 4A). We then examined the impacts of BCKAs on isolated liver mitochondria. Interestingly, BCKAs significantly inhibited Complex II but not Complex I mediated respiration in isolated liver mitochondria (Figures 7B–E), different from the impacts on heart mitochondria (Supplementary Figure S2; Sun et al., 2016). These results indicate that accumulated BCKAs due to BCAA catabolic defect may directly inhibit TCA cycle and mitochondrial oxidative phosphorylation in liver mitochondria.

The TCA cycle intermediates showed no alterations in PP2Cm deficient skeletal muscle (Supplementary Figure S3A). Citrate and *cis*-aconitate showed trends of increase in PP2Cm deficient adipose tissue compared to the wildtype counterpart (Supplementary Figure S3B), although this trend may primarily arise from an outlier data point.

## Discussion

In the present study, we investigated the impacts of BCAA catabolic defect on glucose metabolism in lean mice. Compared to wildtype mice on normal chow, the PP2Cm deficient mice with genetic defect in BCAA catabolism were leaner and favored carbohydrate over lipids for energy production, accompanied with enhanced glucose and insulin tolerance. Metabolomics profiling revealed distinct global metabolic patterns in PP2Cm deficient mice, characterized with tissue-specific BCAA catabolic defects and alterations in gluconeogenesis/glycolysis, glycogen metabolism, and TCA cycle. The predominant impacts of BCAA catabolic defect on glucose metabolism were observed in liver where BCAA catabolic defect led to the accumulation of BCKAs but not BCAAs. Interestingly, BCKAs regulated the gene expression of some key regulators of glucose metabolism and inhibited the succinate dehydrogenase/respiratory Complex II in isolated liver mitochondria, interrupting TCA cycle and mitochondrial oxidative phosphorylation. Together, these results clearly demonstrated the multiple impacts of BCAA catabolic defect on glucose metabolism in lean mice.

PP2Cm is a member of PP2C family and specific phosphatase of BCKD (Lu et al., 2009). Many metabolic phenotypes of PP2Cm deficient mice, such as lower body weight and enhanced insulin sensitivity, are similar to those of mice with BCAA dietary supplement or BCATm ablation (She et al., 2007a; Lynch and Adams, 2014). BCATm is the first enzyme in BCAA degradation pathway to catalyze the conversion of BCAA to BCKA. Ablation of BCATm leads to BCAA catabolic defect and dramatic BCAA accumulation. The similar phenotypes of these different BCAA experimental models strongly suggest that the altered glucose metabolism in PP2Cm deficient mice is caused by the BCAA catabolic defect. A recent study suggests PP2Cm and BCKDK also regulate the activity of a key lipogenic enzyme ATP-citrate lyase (ACL), showing BCKDK increases ACL phosphorylation and lipogenesis (White et al., 2018). Based on this observation, it can be speculated that PP2Cm ablation would enhance lipogenesis (White et al., 2018). However, the PP2Cm deficient mice are leaner compared with the wildtype control. In addition, the plasma levels of some free fatty acids, including stearate, are lower in PP2Cm deficient mice compared with those in control (data not shown). Although the biochemical processes of lipogenesis haven’t been investigated in the current study, it is less likely that there is increased lipogenesis when the PP2Cm deficient mice are leaner. The details warrant further investigation.

Using metabolomics profiling, this study analyzed hundreds of metabolites involving in numerous major metabolic pathways in a high-throughput manner, generating a global view of the metabolic state of the PP2Cm deficient mice. One limitation of current metabolomics analyses is the limited number of metabolites. An analysis of more metabolites will certainly provide extra information for the metabolic alterations. Another limitation is the metabolomics results demonstrated the relative concentrations of metabolites, which can be further validated quantitatively. In the meanwhile, the relative plasma BCAA and BCKA levels in these metabolomics results corroborated the previous studies in which BCAA and BCKA levels were measured quantitatively (Lu et al., 2009). Finally, more mechanistic studies and dynamics metabolomics profiling of PP2Cm deficient mice following meal (or BCAA intake) would provide more insights into the metabolic alterations in PP2Cm deficient mice.

Liver, skeletal muscle, and adipose tissue contribute significantly to the whole body BCAA homeostasis, coordinating via the interorgan shuttling of BCAA and their metabolites (Hutson et al., 2005). Consistent with the global impairment of BCKD activity, BCAA and BCAA catabolic products formed upstream of BCKD are elevated in the plasma of PP2Cm deficient mice. Interestingly, some derivatives from products downstream of BCKD are also increased in PP2Cm deficient plasma, possibly resulting from the interorgan shuttling of BCAA catabolites. BCAAs are elevated in skeletal muscle and adipose tissue but not liver in the PP2Cm deficient mice. In contrast, BCKAs are significantly accumulated in skeletal muscle and liver in the PP2Cm deficient mice. The BCKD downstream catabolites are diminished in PP2Cm deficient skeletal muscle and white adipose tissues. In PP2Cm deficient liver, however, BCKD downstream catabolites are either elevated or diminished, indicating possible influence from the blood. Those data demonstrate great complexity in BCAA catabolism.

The major impacts of BCAA catabolic defect on glucose metabolism have been observed in liver but not adipose tissue or skeletal muscle. Liver is the key organ controlling global glucose metabolism via multiple metabolic pathways including, but not limited to, glycolysis and oxidation, gluconeogenesis, glycogenolysis and glycogenesis. Our data suggest that glycogen metabolism is enhanced in the PP2Cm deficient liver. The elevated glycogen catabolite levels may reflect increased degradation to replenish glucose and facilitate glycolytic metabolism in PP2Cm deficient liver, which is further supported by the elevated upstream glycolytic metabolites (glucose, glucose 6-phosphate and fructose 6-phosphate) in the PP2Cm deficient liver. Interestingly, the downstream glycolytic intermediates (fructose-1,6-biphosphate, 3-phosphoglycerate) are lower in the PP2Cm deficient liver. Since the upstream glycolytic metabolites are important compounds at the junction of several other metabolic pathways including the pentose phosphate pathway and the polyol pathway, it is possible that the elevated upstream metabolites in glycolysis enter these pathways, leading to the lower downstream metabolites.

Branched-chain keto acids may play a unique role in glucose metabolic regulation in liver. Higher BCKA, but not BCAA, abundances are detected in the PP2Cm deficient liver. BCKAs, but not BCAAs, markedly increases PYGL expression in HepG2 cell, consistent with the higher PYGL expression in PP2Cm deficient liver and indicating a direct regulation on glycogen metabolism by BCKAs. On the other hand, the metabolomics profile of TCA cycle in PP2Cm deficient liver is consistent with the inhibitory effects of BCKAs on respiration Complex II/succinate dehydrogenase in isolated mitochondria. Interestingly, these impacts on liver glucose metabolism are not observed in PP2Cm deficient skeletal muscle or adipose tissue even BCKAs are elevated in these tissues. Thus, BCAA catabolism defect may affect glucose metabolism via BCKA accumulation in liver.

In the PP2Cm deficient mice, BCAA catabolic defect enhances insulin sensitivity in lean animals. Similarly, in another genetic model, inactivation of mitochondrial BCAT abolishes BCAA catabolism, resulting in lower adiposity and improved glucose tolerance in lean mice (She et al., 2007a). These beneficial impacts of BCAA catabolic defect on insulin sensitivity are opposite to its stimulatory effects on insulin resistance in obese mice, supporting the notion that BCAAs exert different metabolic effects depending on the catabolic and anabolic states (Bifari and Nisoli, 2017). The underlying mechanisms remain unclear. It has been suggested that tissue-specific alterations of BCAA catabolic flux may play a role in the obesity-associated insulin resistance. The reduced catabolism by some tissues, such as adipose tissue, shunts the BCAA oxidation toward other tissues, such as skeletal muscle, in obese mice, and promotes insulin resistance (Newgard, 2012; Neinast et al., 2018). In the genetic models of PP2Cm or BCATm knockout mice, however, the BCAA catabolism is likely impaired in all tissues. These observations indicate a critical role of the BCAA catabolism in skeletal muscle in determining insulin sensitivity. On the other hand, the reduced body weight (Figure 1A), adiposity, and white adipose mass (data not shown) in the lean PP2Cm KO mice may contribute to the enhanced insulin sensitivity. In addition, numerous studies have indicated that BCAA and BCKA can directly regulate pyruvate dehydrogenase complex activity (Li et al., 2017), mitochondrial respiration (Figure 7) (Sun et al., 2016), and insulin signaling and secretion (Lynch and Adams, 2014), which may all affect glucose metabolism in lean mice.

In summary, the current study shows BCAA catabolic defect affects glucose metabolism in lean mice. BCAA catabolic defect leads lower body weight and better glucose tolerance. These beneficial impacts are consistent with previous reports showing BCAA supplementation or BCAA-rich protein diets are associated with positive effects on body weight and glucose homeostasis (Lynch and Adams, 2014). On the other hand, recent studies show BCKD expression is reduced in obese and diabetic animals and humans, likely leading to BCAA catabolic defect and contributing to the development of insulin resistance and diabetes (She et al., 2007b, 2013; Pietiläinen et al., 2008; Herman et al., 2010; Lackey et al., 2013; Menni et al., 2013; Zimmerman et al., 2013; Cummings et al., 2018; White et al., 2018). Thus, the BCAA catabolic defect may show different even opposite impacts on glucose metabolism in lean and obese animals. While this discrepancy has been noted recently and the mechanisms remain unclear (Bifari and Nisoli, 2017), our results highlight the critical role of BCAA catabolism in glycemic regulation and the complex interplay among macronutrients.

## Data Availability

The datasets generated for this study are available on request to the corresponding author.

## Ethics Statement

Animal Subjects: The animal study was reviewed and approved by Committee for Humane Treatment of Animals at Shanghai Jiao Tong University School of Medicine or the University of California at Los Angeles Institutional Animal Care and Use Committee.

## Author Contributions

HS and MZ designed the research. JW, YL, XS, JS, and MC performed the research. YL, JW, KL, JF, MZ, and HS analyzed the data. YW helped to design the overall study and analyzed the data. HS, MZ, JW, and KL contributed to the manuscript preparation.

## Conflict of Interest Statement

HS and YW participate in an advisory board for Ramino Bio Ltd. The remaining authors declare that the research was conducted in the absence of any commercial or financial relationships that could be construed as a potential conflict of interest.

## Abbreviations

BCAA: branched-chain amino acid
BCAT: BCAA transaminase
BCKA: branched-chain keto acid
BCKD: branched-chain- α-ketoacid dehydrogenase
BCKDK: branched-chain- α-ketoacid dehydrogenase kinase
GTT: glucose tolerance test
ITT: insulin tolerance test
PP2Cm: mitochondrial protein phosphatase 2C
PPm1k: protein phosphatase 1K (PP2C domain containing)
RER: respiratory exchange ratio
ROS: reactive oxygen species
T2DM: type 2 diabetes mellitus
TCA cycle: tricarboxylic acid cycle
