# Supplementary figures and images for: BCAA Catabolic Defect Alters Glucose Metabolism in Lean Mice
Source: Front Physiol. 2019 Sep 4;10:1140. doi: 10.3389/fphys.2019.01140 (PMC6738029; doi:10.3389/fphys.2019.01140)

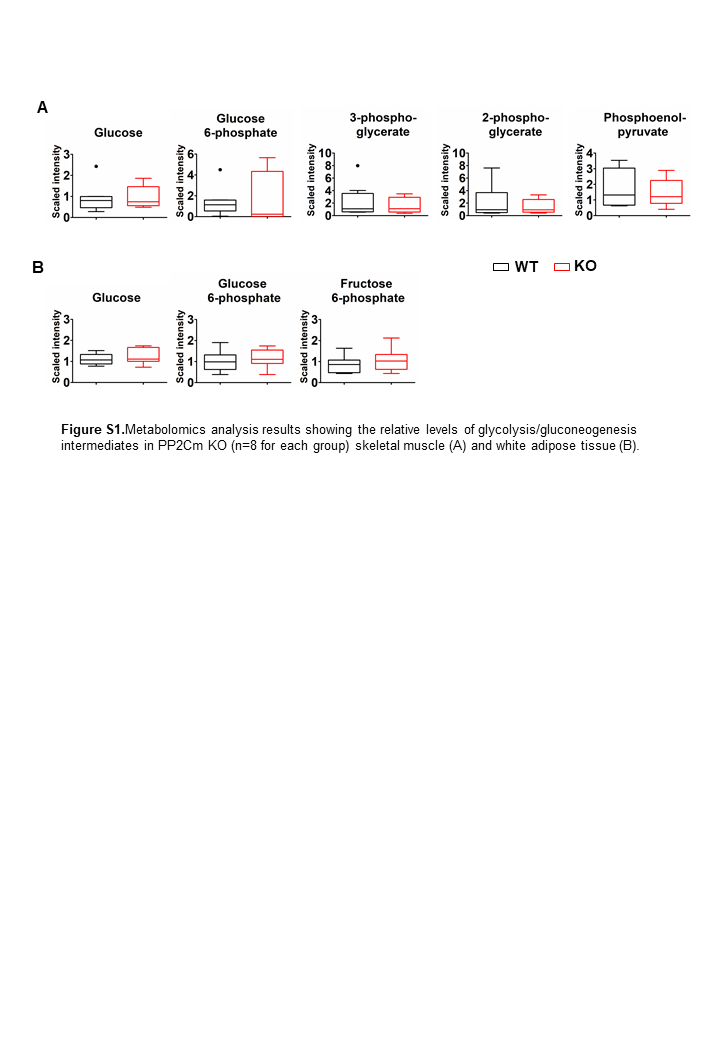

Supplement: Supplementary file 1 [file Image_1.TIF]

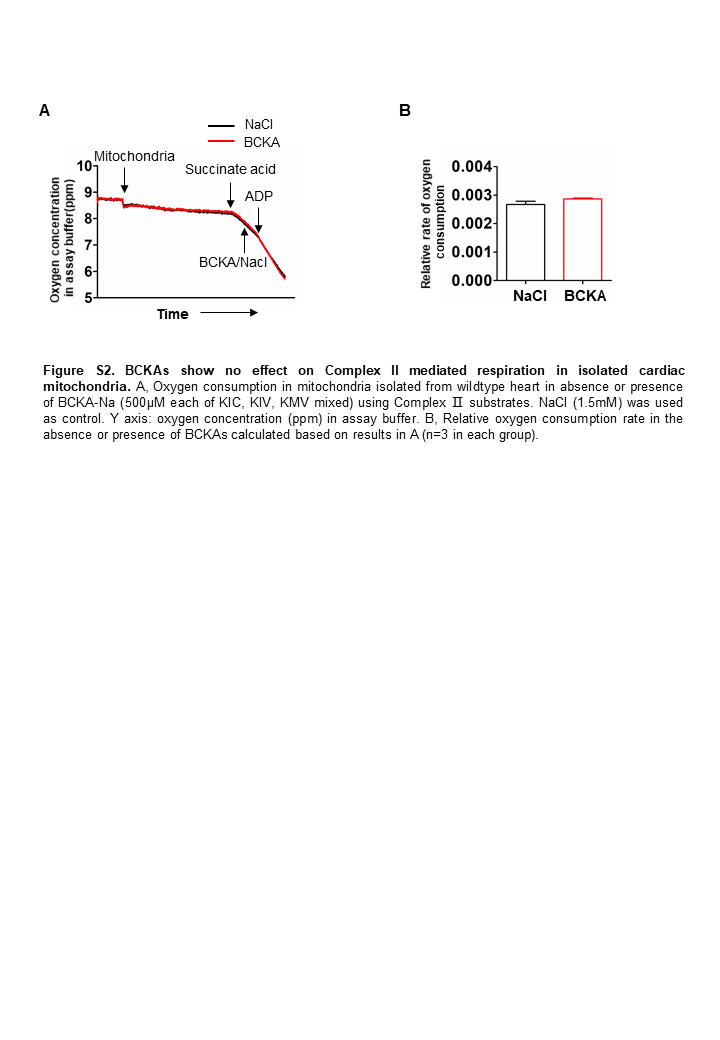

Supplement: Supplementary file 2 [file Image_2.TIF]

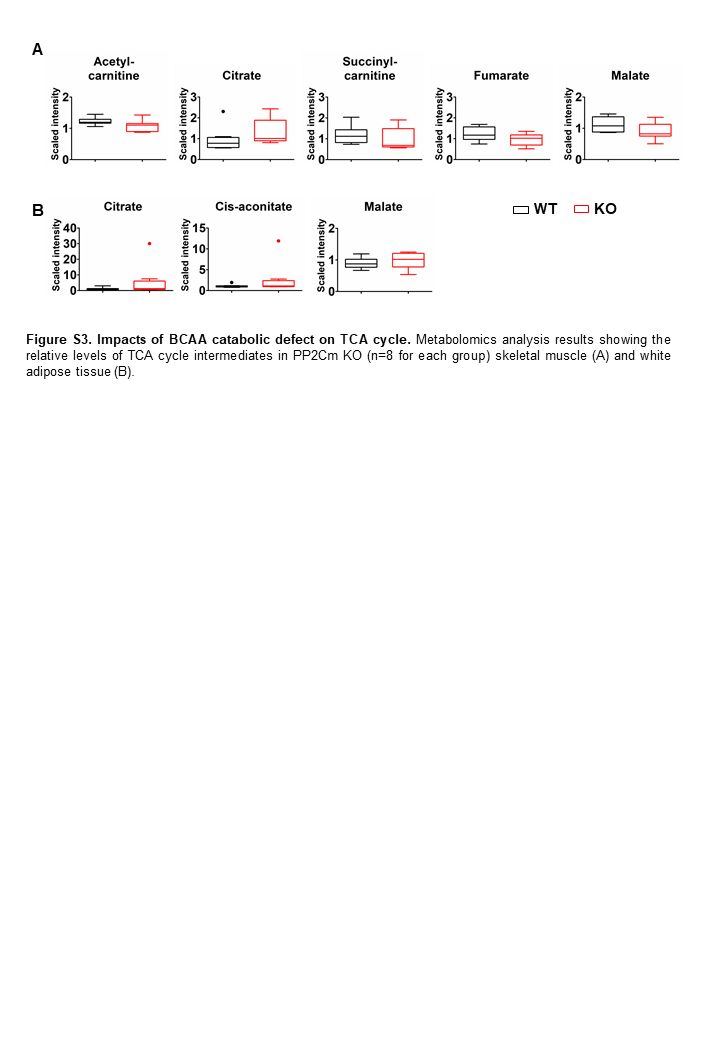

Supplement: Supplementary file 3 [file Image_3.TIF]
