# Supplementary material for: BCAA Catabolic Defect Alters Glucose Metabolism in Lean Mice
Source: Front Physiol. 2019 Sep 4;10:1140. doi: 10.3389/fphys.2019.01140 (PMC6738029; doi:10.3389/fphys.2019.01140)
Supplement: Supplementary file 4 [file Table_1.pdf]

Supplementary Table 1 : Metabolites in Plasma

BIOCHEMICAL	SUPER PATHWAY
1-(3-aminopropyl)-2-pyrrolidone	Xenobiotics
1,2-propanediol	Xenobiotics
1,3-dihydroxyacetone	Carbohydrate
1,5-anhydroglucitol (1,5-AG)	Carbohydrate
10-heptadecenoate (17:1n7)	Lipid
10-nonadecenoate (19:1n9)	Lipid
10-undecenoate (11:1n1)	Lipid
12,13-DiHOME	Lipid
12-HETE	Lipid
13-HODE + 9-HODE	Lipid
15-methylpalmitate (isobar with 2-methylpalmitate)	Lipid
17-methylstearate	Lipid
1-arachidonoylglycerophosphocholine (20:4n6)*	Lipid
1-arachidonoylglycerophosphoethanolamine*	Lipid
1-arachidonoylglycerophosphoinositol*	Lipid
1-arachidoylglycerophosphocholine (20:0)	Lipid
1-dihomo-linoleoylglycerophosphocholine (20:2n6)*	Lipid
1-docosahexaenoylglycerophosphocholine (22:6n3)*	Lipid
1-docosahexaenoylglycerophosphoethanolamine*	Lipid
1-docosapentaenoylglycerophosphocholine (22:5n3)*	Lipid
1-eicosapentaenoylglycerophosphocholine (20:5n3)*	Lipid
1-eicosatrienoylglycerophosphocholine (20:3)*	Lipid
1-eicosenoylglycerophosphocholine (20:1n9)*	Lipid
1-linolenoylglycerophosphocholine (18:3n3)*	Lipid
1-linoleoylglycerol (1-monolinolein)	Lipid
1-linoleoylglycerophosphocholine (18:2n6)	Lipid
1-linoleoylglycerophosphoethanolamine*	Lipid
1-linoleoylglycerophosphoinositol*	Lipid
1-margaroylglycerophosphocholine (17:0)	Lipid
1-methylimidazoleacetate	Amino Acid
1-myristoylglycerophosphocholine (14:0)	Lipid
1-nonadecanoylglycerophosphocholine(19:0)*	Lipid
1-oleoylglycerol (1-monoolein)	Lipid
1-oleoylglycerophosphocholine (18:1)	Lipid
1-oleoylglycerophosphoethanolamine	Lipid
1-palmitoleoylglycerophosphocholine (16:1)*	Lipid
1-palmitoylglycerol (1-monopalmitin)	Lipid
1-palmitoylglycerophosphocholine (16:0)	Lipid
1-palmitoylglycerophosphoethanolamine	Lipid
1-palmitoylglycerophosphoinositol*	Lipid
1-palmitoylplasmenylethanolamine*	Lipid
1-pentadecanoylglycerophosphocholine (15:0)*	Lipid
1-stearoylglycerophosphocholine (18:0)	Lipid
1-stearoylglycerophosphoethanolamine	Lipid
1-stearoylglycerophosphoinositol	Lipid
2-aminobutyrate	Amino Acid
2-aminoheptanoate	Lipid
2-aminooctanoate	Lipid
2-arachidonoylglycerophosphocholine*	Lipid
2-arachidonoylglycerophosphoethanolamine*	Lipid
2'-deoxycytidine	Nucleotide
2'-deoxyuridine	Nucleotide
2-docosahexaenoylglycerophosphocholine*	Lipid
2-docosahexaenoylglycerophosphoethanolamine*	Lipid
2-hydroxy-3-methylvalerate	Amino Acid

2-hydroxybutyrate (AHB)	Amino Acid
2-hydroxydecanoate	Lipid
2-hydroxyglutarate	Lipid
2-hydroxyisobutyrate	Xenobiotics
2-hydroxypalmitate	Lipid
2-hydroxystearate	Lipid
2-linoleoylglycerophosphocholine*	Lipid
2-linoleoylglycerophosphoethanolamine*	Lipid
2-methylbutyrylcarnitine (C5)	Amino Acid
2-myristoylglycerophosphocholine*	Lipid
2-oleoylglycerophosphocholine*	Lipid
2-oleoylglycerophosphoethanolamine*	Lipid
2-palmitoleoylglycerophosphocholine*	Lipid
2-palmitoylglycerophosphocholine*	Lipid
2-palmitoylglycerophosphoethanolamine*	Lipid
2-stearoylglycerophosphocholine*	Lipid
3-(4-hydroxyphenyl)lactate	Amino Acid
3-dehydrocarnitine*	Lipid
3-hydroxybutyrate (BHBA)	Lipid
3-hydroxydecanoate	Lipid
3-hydroxyoctanoate	Lipid
3-hydroxypropanoate	Lipid
3-indoxyl sulfate	Amino Acid
3-methyl-2-oxobutyrate	Amino Acid
3-methyl-2-oxovalerate	Amino Acid
3-methylcrotonylglycine	Amino Acid
3-methylglutarylcarnitine (C6)	Amino Acid
3-phenylpropionate (hydrocinnamate)	Amino Acid
3-ureidopropionate	Nucleotide
4-ethylphenylsulfate	Xenobiotics
4-guanidinobutanoate	Amino Acid
4-hydroxycinnamate	Amino Acid
4-hydroxyphenylpyruvate	Amino Acid
4-methyl-2-oxopentanoate	Amino Acid
4-vinylphenol sulfate	Xenobiotics
5,6-dihydrouracil	Nucleotide
5-dodecenoate (12:1n7)	Lipid
5-methylthioadenosine (MTA)	Amino Acid
5-oxoproline	Amino Acid
7-alpha-hydroxy-3-oxo-4-cholestenoate (7-Hoca)	Lipid
acetoacetate	Lipid
acetylcarnitine	Lipid
adenosine	Nucleotide
adenosine 5'-monophosphate (AMP)	Nucleotide
adrenate (22:4n6)	Lipid
alanine	Amino Acid
alanylalanine	Peptide
allantoin	Nucleotide
alpha-hydroxyisocaproate	Amino Acid
alpha-hydroxyisovalerate	Amino Acid
alpha-ketoglutarate	Energy
alpha-tocopherol	Cofactors and Vitamins
arabinose	Carbohydrate
arabitol	Carbohydrate
arachidate (20:0)	Lipid
arachidonate (20:4n6)	Lipid
arginine	Amino Acid
asparagine	Amino Acid

beta-alanine	Nucleotide
beta-hydroxyisovaleroylcarnitine	Amino Acid
beta-hydroxypyruvate	Amino Acid
betaine	Amino Acid
beta-muricholate	Lipid
beta-sitosterol	Lipid
bradykinin, des-arg(9)	Peptide
butyrylcarnitine	Lipid
butyrylglycine	Lipid
campesterol	Lipid
caprate (10:0)	Lipid
caproate (6:0)	Lipid
caprylate (8:0)	Lipid
carnitine	Lipid
catechol sulfate	Xenobiotics
C-glycosyltryptophan*	Amino Acid
chiro-inositol	Lipid
cholate	Lipid
cholestanol	Lipid
cholesterol	Lipid
choline	Lipid
cinnamoylglycine	Xenobiotics
cis-aconitate	Energy
cis-vaccenate (18:1n7)	Lipid
citrate	Energy
citrulline	Amino Acid
corticosterone	Lipid
creatine	Amino Acid
creatinine	Amino Acid
cysteine	Amino Acid
cysteine-glutathione disulfide	Amino Acid
cystine	Amino Acid
cytidine	Nucleotide
daidzein	Xenobiotics
deoxycarnitine	Lipid
deoxycholate	Lipid
dihomo-linoleate (20:2n6)	Lipid
dihomo-linolenate (20:3n3 or n6)	Lipid
dimethylglycine	Amino Acid
docosadienoate (22:2n6)	Lipid
docosadioate	Lipid
docosahexaenoate (DHA; 22:6n3)	Lipid
docosapentaenoate (n3 DPA; 22:5n3)	Lipid
docosapentaenoate (n6 DPA; 22:5n6)	Lipid
eicosapentaenoate (EPA; 20:5n3)	Lipid
eicosenoate (20:1n9 or 11)	Lipid
equol glucuronide	Xenobiotics
equol sulfate	Xenobiotics
erythritol	Xenobiotics
erythronate*	Carbohydrate
ethyl glucuronide	Xenobiotics
fructose	Carbohydrate
fucose	Carbohydrate
fumarate	Energy
gamma-glutamylalanine	Peptide
gamma-glutamylglycine	Peptide
gamma-glutamylisoleucine*	Peptide
gamma-glutamylleucine	Peptide

gamma-glutamylmethionine	Peptide
gamma-glutamylphenylalanine	Peptide
gamma-glutamyltyrosine	Peptide
gamma-glutamylvaline	Peptide
glucose	Carbohydrate
glucose-6-phosphate (G6P)	Carbohydrate
glutamate	Amino Acid
glutamine	Amino Acid
glutathione, oxidized (GSSG)	Amino Acid
glycerate	Carbohydrate
glycerol	Lipid
glycerol 2-phosphate	Xenobiotics
glycerol 3-phosphate (G3P)	Lipid
glycerophosphorylcholine (GPC)	Lipid
glycine	Amino Acid
glycolate (hydroxyacetate)	Xenobiotics
gulono-1,4-lactone	Cofactors and Vitamins
heme	Cofactors and Vitamins
hexadecanedioate	Lipid
hexanoylcarnitine	Lipid
hexanoylglycine	Lipid
hippurate	Xenobiotics
histidine	Amino Acid
homostachydrine*	Xenobiotics
hydroxybutyrylcarnitine*	Lipid
hypoxanthine	Nucleotide
indolelactate	Amino Acid
indolepropionate	Amino Acid
inosine	Nucleotide
inositol 1-phosphate (I1P)	Lipid
isobutyrylcarnitine	Amino Acid
isocaproate	Lipid
isoleucine	Amino Acid
isovalerate	Amino Acid
isovalerylcarnitine	Amino Acid
isovalerylglycine	Amino Acid
kynurenate	Amino Acid
kynurenine	Amino Acid
lactate	Carbohydrate
laurylcarnitine	Lipid
leucine	Amino Acid
linoleate (18:2n6)	Lipid
linolenate [alpha or gamma; (18:3n3 or 6)]	Lipid
lysine	Amino Acid
malate	Energy
mannitol	Carbohydrate
mannose	Carbohydrate
margarate (17:0)	Lipid
mead acid (20:3n9)	Lipid
metacryloyl glycine	Xenobiotics
methionine	Amino Acid
methyl-alpha-glucopyranoside	Xenobiotics
methyl-beta-glucopyranoside	Carbohydrate
myo-inositol	Lipid
myristate (14:0)	Lipid
myristoleate (14:1n5)	Lipid
myristoylcarnitine	Lipid
N1-Methyl-2-pyridone-5-carboxamide	Cofactors and Vitamins

N1-methyladenosine	Nucleotide
N1-methylguanosine	Nucleotide
N-acetylalanine	Amino Acid
N-acetylaspartate (NAA)	Amino Acid
N-acetyl-beta-alanine	Nucleotide
N-acetylglycine	Amino Acid
N-acetylisoleucine	Amino Acid
N-acetylleucine	Amino Acid
N-acetylmethionine	Amino Acid
N-acetylneuraminate	Carbohydrate
N-acetylphenylalanine	Amino Acid
N-acetyltryptophan	Amino Acid
N-acetyltyrosine	Amino Acid
N-delta-acetylornithine*	Amino Acid
nicotinamide	Cofactors and Vitamins
N-octanoylglycine	Lipid
nonadecanoate (19:0)	Lipid
octanoylcarnitine	Lipid
oleate (18:1n9)	Lipid
ophthalmate	Amino Acid
ornithine	Amino Acid
palmitate (16:0)	Lipid
palmitoleate (16:1n7)	Lipid
palmitoyl ethanolamide	Lipid
palmitoyl sphingomyelin	Lipid
palmitoylcarnitine	Lipid
pantothenate	Cofactors and Vitamins
p-cresol sulfate	Amino Acid
pelargonate (9:0)	Lipid
phenol sulfate	Amino Acid
phenylacetate	Amino Acid
phenylacetylglycine	Amino Acid
phenylalanine	Amino Acid
phenyllactate (PLA)	Amino Acid
phenylpropionylglycine	Amino Acid
phosphate	Energy
pinitol	Lipid
pipecolate	Amino Acid
pro-hydroxy-pro	Amino Acid
proline	Amino Acid
propionylcarnitine	Lipid
pseudouridine	Nucleotide
pyridoxate	Cofactors and Vitamins
pyrophosphate (PPi)	Energy
pyruvate	Carbohydrate
riboflavin (Vitamin B2)	Cofactors and Vitamins
ribose	Carbohydrate
serine	Amino Acid
serotonin (5HT)	Amino Acid
S-methylcysteine	Amino Acid
sorbitol	Carbohydrate
sphinganine	Lipid
stachydrine	Xenobiotics
stearate (18:0)	Lipid
stearidonate (18:4n3)	Lipid
stearoyl sphingomyelin	Lipid
stearoylcarnitine	Lipid
succinate	Energy

succinylcarnitine	Energy
tartarate	Xenobiotics
taurine	Amino Acid
tauro(alpha + beta)muricholate	Lipid
taurochenodeoxycholate	Lipid
taurocholate	Lipid
taurodeoxycholate	Lipid
taurohyodeoxycholic acid	Lipid
tauroursodeoxycholate	Lipid
TDTEDKGEFLSEGGGV*	Peptide
TDTEDKGEFLSEGGGVR*	Peptide
threonate	Cofactors and Vitamins
threonine	Amino Acid
thymidine	Nucleotide
tigloylglycine	Amino Acid
trans-4-hydroxyproline	Amino Acid
trans-urocanate	Amino Acid
tryptophan	Amino Acid
tyrosine	Amino Acid
uracil	Nucleotide
urate	Nucleotide
urea	Amino Acid
uridine	Nucleotide
valerylcarnitine	Lipid
valerylglycine	Lipid
valine	Amino Acid
xanthine	Nucleotide
xylose	Carbohydrate
