# Supplementary material for: BCAA Catabolic Defect Alters Glucose Metabolism in Lean Mice
Source: Front Physiol. 2019 Sep 4;10:1140. doi: 10.3389/fphys.2019.01140 (PMC6738029; doi:10.3389/fphys.2019.01140)
Supplement: Supplementary file 5 [file Table_2.pdf]

Supplementary Table 2： Metabolites in Liver

BIOCHEMICAL	SUPER_PATHWAY
1,2-dipalmitoylglycerol	Lipid
1,5-anhydroglucitol (1,5-AG)	Carbohydrate
1-arachidonoylglycerophosphocholine (20:4n6)*	Lipid
1-arachidonoylglycerophosphoethanolamine*	Lipid
1-arachidonoylglycerophosphoinositol*	Lipid
1-linoleoylglycerophosphocholine (18:2n6)	Lipid
1-linoleoylglycerophosphoethanolamine*	Lipid
1-methylimidazoleacetate	Amino Acid
1-oleoylglycerol (1-monoolein)	Lipid
1-oleoylglycerophosphocholine (18:1)	Lipid
1-oleoylglycerophosphoethanolamine	Lipid
1-oleoylglycerophosphoinositol*	Lipid
1-palmitoleoylglycerophosphocholine (16:1)*	Lipid
1-palmitoylglycerol (1-monopalmitin)	Lipid
1-palmitoylglycerophosphocholine (16:0)	Lipid
1-palmitoylglycerophosphoethanolamine	Lipid
1-palmitoylglycerophosphoglycerol*	Lipid
1-palmitoylglycerophosphoinositol*	Lipid
1-palmitoylglycerophosphoserine*	Lipid
1-palmitoylplasmenylethanolamine*	Lipid
1-stearoylglycerol (1-monostearin)	Lipid
1-stearoylglycerophosphocholine (18:0)	Lipid
1-stearoylglycerophosphoethanolamine	Lipid
1-stearoylglycerophosphoglycerol	Lipid
1-stearoylglycerophosphoinositol	Lipid
10-heptadecenoate (17:1n7)	Lipid
10-nonadecenoate (19:1n9)	Lipid
12,13-DiHOME	Lipid
12-HEPE	Lipid
12-HETE	Lipid
13-HODE + 9-HODE	Lipid
15-HETE	Lipid
15-methylpalmitate (isobar with 2-methylpalmitate)	Lipid
16-hydroxypalmitate	Lipid
17-methylstearate	Lipid
2'-deoxyinosine	Nucleotide
2-aminoadipate	Amino Acid
2-aminobutyrate	Amino Acid
2-aminoheptanoate	Lipid
2-hydroxybutyrate (AHB)	Amino Acid
2-hydroxyglutarate	Lipid
2-hydroxystearate	Lipid
2-methylbutyrylcarnitine (C5)	Amino Acid
2-palmitoleoylglycerophosphocholine*	Lipid
2-palmitoylglycerol (2-monopalmitin)	Lipid
2-palmitoylglycerophosphocholine*	Lipid
2-stearoylglycerophosphocholine*	Lipid
3'-dephosphocoenzyme A	Cofactors and Vitamins
3-(4-hydroxyphenyl)lactate	Amino Acid
3-aminobutyrate	Xenobiotics
3-aminoisobutyrate	Nucleotide
3-hydroxybutyrate (BHBA)	Lipid
3-hydroxybutyryl CoA	Lipid
3-hydroxydecanoate	Lipid
3-indoxyl sulfate	Amino Acid

3-methyl-2-oxovalerate	Amino Acid
3-methylglutarylcarnitine (C6)	Amino Acid
3-phosphoglycerate	Carbohydrate
3-ureidopropionate	Nucleotide
4-guanidinobutanoate	Amino Acid
4-hydroxy-nonenal-glutathione	Amino Acid
4-hydroxybutyrate (GHB)	Lipid
4-methyl-2-oxopentanoate	Amino Acid
5-aminovalerate	Amino Acid
5-methyltetrahydrofolate (5MeTHF)	Cofactors and Vitamins
5-methylthioadenosine (MTA)	Amino Acid
5-oxoproline	Amino Acid
6-beta-hydroxylithocholate	Lipid
6-phosphogluconate	Carbohydrate
7-alpha-hydroxy-3-oxo-4-cholestenoate (7-Hoca)	Lipid
acetylcarnitine	Lipid
adenine	Nucleotide
adenosine	Nucleotide
adenosine 2'-monophosphate (2'-AMP)	Nucleotide
adenosine 3',5'-diphosphate	Nucleotide
adenosine 3'-monophosphate (3'-AMP)	Nucleotide
adenosine 5'-diphosphate (ADP)	Nucleotide
adenosine 5'-diphosphoribose (ADP-ribose)	Cofactors and Vitamins
adenosine 5'-monophosphate (AMP)	Nucleotide
adenylosuccinate	Nucleotide
adrenate (22:4n6)	Lipid
alanine	Amino Acid
allantoin	Nucleotide
allo-isoleucine	Amino Acid
alpha-hydroxyisovalerate	Amino Acid
alpha-muricholate	Lipid
alpha-tocopherol	Cofactors and Vitamins
arabitol	Carbohydrate
arabonate	Cofactors and Vitamins
arachidate (20:0)	Lipid
arachidonate (20:4n6)	Lipid
ascorbate (Vitamin C)	Cofactors and Vitamins
asparagine	Amino Acid
aspartate	Amino Acid
azelate (nonanedioate)	Lipid
beta-alanine	Nucleotide
beta-hydroxyisovaleroylcarnitine	Amino Acid
beta-muricholate	Lipid
betaine	Amino Acid
biopterin	Cofactors and Vitamins
butyrylcarnitine	Lipid
butyrylglycine	Lipid
C-mannosyltryptophan	Amino Acid
campesterol	Lipid
carnitine	Lipid
cholate	Lipid
cholestanol	Lipid
cholesterol	Lipid
choline	Lipid
choline phosphate	Lipid
cinnamoylglycine	Xenobiotics
cis-vaccenate (18:1n7)	Lipid
citrate	Energy

citrulline	Amino Acid
coenzyme A	Cofactors and Vitamins
coprostanol	Lipid
corticosterone	Lipid
creatine	Amino Acid
creatinine	Amino Acid
cysteine	Amino Acid
cysteine-glutathione disulfide	Amino Acid
cytidine	Nucleotide
cytidine 5'-diphosphocholine	Lipid
cytidine 5'-monophosphate (5'-CMP)	Nucleotide
cytidine-5'-diphosphoethanolamine	Lipid
dehydroascorbate	Cofactors and Vitamins
deoxycarnitine	Lipid
dihomo-linoleate (20:2n6)	Lipid
dihomo-linolenate (20:3n3 or n6)	Lipid
dihydrobiopterin	Cofactors and Vitamins
dihydroxyacetone phosphate (DHAP)	Carbohydrate
dimethylarginine (SDMA + ADMA)	Amino Acid
dimethylglycine	Amino Acid
docosadienoate (22:2n6)	Lipid
docosahexaenoate (DHA; 22:6n3)	Lipid
docosapentaenoate (n3 DPA; 22:5n3)	Lipid
docosapentaenoate (n6 DPA; 22:5n6)	Lipid
eicosapentaenoate (EPA; 20:5n3)	Lipid
eicosenoate (20:1n9 or 11)	Lipid
equol	Xenobiotics
equol glucuronide	Xenobiotics
equol sulfate	Xenobiotics
ergothioneine	Xenobiotics
erucate (22:1n9)	Lipid
erythritol	Xenobiotics
erythronate*	Carbohydrate
ethanolamine	Lipid
ethyl glucuronide	Xenobiotics
flavin adenine dinucleotide (FAD)	Cofactors and Vitamins
flavin mononucleotide (FMN)	Cofactors and Vitamins
fructose	Carbohydrate
fructose-6-phosphate	Carbohydrate
fumarate	Energy
gamma-glutamylglutamate	Peptide
gamma-glutamylisoleucine*	Peptide
gamma-glutamylleucine	Peptide
gamma-glutamyltyrosine	Peptide
gamma-glutamylvaline	Peptide
gluconate	Xenobiotics
glucosamine	Carbohydrate
glucose	Carbohydrate
glucose-6-phosphate (G6P)	Carbohydrate
glucuronate	Carbohydrate
glutamate	Amino Acid
glutamate, gamma-methyl ester	Amino Acid
glutamine	Amino Acid
glutarylcarnitine (C5)	Amino Acid
glutathione, oxidized (GSSG)	Amino Acid
glutathione, reduced (GSH)	Amino Acid
glycerate	Carbohydrate
glycerol	Lipid

glycerol 3-phosphate (G3P)	Lipid
glycerophosphorylcholine (GPC)	Lipid
glycine	Amino Acid
glycocholate	Lipid
glycylisoleucine	Peptide
glycylleucine	Peptide
glycylvaline	Peptide
guanosine	Nucleotide
guanosine 5'- monophosphate (5'-GMP)	Nucleotide
heme	Cofactors and Vitamins
hexadecanedioate	Lipid
hexanoylglycine	Lipid
hexenedioylcarnitine*	Lipid
hippurate	Xenobiotics
histidine	Amino Acid
homocitrulline	Amino Acid
homoserine	Amino Acid
homostachydrine*	Xenobiotics
hydroxybutyrylcarnitine*	Lipid
hypotaurine	Amino Acid
hypoxanthine	Nucleotide
imidazole propionate	Amino Acid
inosine	Nucleotide
inosine 5'-monophosphate (IMP)	Nucleotide
inositol 1-phosphate (I1P)	Lipid
Isobar: betaine aldehyde, N-methyldiethanolamine	Amino Acid
Isobar: fructose 1,6-diphosphate, glucose 1,6-diphosphate, my	Carbohydrate
isobutyrylcarnitine	Amino Acid
isoleucine	Amino Acid
isoleucylglycine	Peptide
isovalerylcarnitine	Amino Acid
isovalerylglycine	Amino Acid
kynurenine	Amino Acid
lactate	Carbohydrate
leucine	Amino Acid
linoleate (18:2n6)	Lipid
linolenate [alpha or gamma; (18:3n3 or 6)]	Lipid
lysine	Amino Acid
malate	Energy
maltohexaose	Carbohydrate
maltopentaose	Carbohydrate
maltose	Carbohydrate
maltotetraose	Carbohydrate
maltotriose	Carbohydrate
mannose	Carbohydrate
mannose-6-phosphate	Carbohydrate
margarate (17:0)	Lipid
mead acid (20:3n9)	Lipid
methionine	Amino Acid
methionine sulfoxide	Amino Acid
methylphosphate	Nucleotide
myo-inositol	Lipid
myristate (14:0)	Lipid
myristoleate (14:1n5)	Lipid
N-acetylalanine	Amino Acid
N-acetylglucosamine 6-phosphate	Carbohydrate
N-acetylglutamate	Amino Acid
N-acetylglutamine	Amino Acid

N-acetylglycine	Amino Acid
N-acetylleucine	Amino Acid
N-acetylmethionine	Amino Acid
N-delta-acetylornithine*	Amino Acid
N-glycolylneuraminate	Xenobiotics
N-octanoylglycine	Lipid
N-oleoyltaurine	Lipid
N-palmitoyltaurine	Lipid
N-stearoyltaurine	Lipid
N1-methyladenosine	Nucleotide
N2-acetyllysine	Amino Acid
N6-acetyllysine	Amino Acid
nicotinamide	Cofactors and Vitamins
nicotinamide adenine dinucleotide (NAD+)	Cofactors and Vitamins
nicotinamide adenine dinucleotide reduced (NADH)	Cofactors and Vitamins
nicotinamide riboside*	Cofactors and Vitamins
nicotinate	Cofactors and Vitamins
nonadecanoate (19:0)	Lipid
oleate (18:1n9)	Lipid
ophthalmate	Amino Acid
ornithine	Amino Acid
p-cresol sulfate	Amino Acid
p-cresol-glucuronide*	Amino Acid
palmitate (16:0)	Lipid
palmitoleate (16:1n7)	Lipid
palmitoyl ethanolamide	Lipid
palmitoyl sphingomyelin	Lipid
palmitoylcarnitine	Lipid
pantothenate	Cofactors and Vitamins
phenol sulfate	Amino Acid
phenylacetylglycine	Amino Acid
phenylalanine	Amino Acid
phosphate	Energy
phosphoethanolamine	Lipid
phosphoglycolate	Xenobiotics
phosphopantetheine	Cofactors and Vitamins
pipecolate	Amino Acid
pro-hydroxy-pro	Amino Acid
proline	Amino Acid
propionylcarnitine	Lipid
prostaglandin F2alpha	Lipid
pseudouridine	Nucleotide
putrescine	Amino Acid
pyridoxal	Cofactors and Vitamins
pyridoxate	Cofactors and Vitamins
pyruvate	Carbohydrate
ribitol	Carbohydrate
riboflavin (Vitamin B2)	Cofactors and Vitamins
ribose	Carbohydrate
ribose 5-phosphate	Carbohydrate
ribulose	Carbohydrate
ribulose/xylulose 5-phosphate	Carbohydrate
S-adenosylhomocysteine (SAH)	Amino Acid
S-adenosylmethionine (SAM)	Amino Acid
S-lactoylglutathione	Amino Acid
S-methylcysteine	Amino Acid
S-methylglutathione	Amino Acid
sarcosine (N-Methylglycine)	Amino Acid

serine	Amino Acid
sorbitol	Carbohydrate
spermidine	Amino Acid
sphinganine	Lipid
sphingosine	Lipid
squalene	Lipid
stachydrine	Xenobiotics
stearate (18:0)	Lipid
stearidonate (18:4n3)	Lipid
stearoyl sphingomyelin	Lipid
stearoylcarnitine	Lipid
succinate	Energy
succinylcarnitine	Energy
tagatose	Carbohydrate
taurine	Amino Acid
tauro(alpha + beta)muricholate	Lipid
taurochenodeoxycholate	Lipid
taurocholate	Lipid
taurodeoxycholate	Lipid
taurohyocholate	Lipid
taurolithocholate	Lipid
tauroursodeoxycholate	Lipid
tetradecanedioate	Lipid
thiamin (Vitamin B1)	Cofactors and Vitamins
thiamin diphosphate	Cofactors and Vitamins
threonate	Cofactors and Vitamins
threonine	Amino Acid
thymidine	Nucleotide
tiglyl carnitine	Amino Acid
trans-4-hydroxyproline	Amino Acid
trigonelline (N'-methylnicotinate)	Cofactors and Vitamins
tryptophan	Amino Acid
tyrosine	Amino Acid
UDP-acetylglucosamine/galactosamine	Carbohydrate
UDP-galactose	Carbohydrate
UDP-glucose	Carbohydrate
UDP-glucuronate	Carbohydrate
undecanedioate	Lipid
uracil	Nucleotide
urea	Amino Acid
uridine	Nucleotide
uridine monophosphate (5' or 3')	Nucleotide
valerylglycine	Lipid
valine	Amino Acid
xanthine	Nucleotide
xanthosine	Nucleotide
xylitol	Carbohydrate
xylonate	Carbohydrate
xylose	Carbohydrate
