# Supplementary material for: BCAA Catabolic Defect Alters Glucose Metabolism in Lean Mice
Source: Front Physiol. 2019 Sep 4;10:1140. doi: 10.3389/fphys.2019.01140 (PMC6738029; doi:10.3389/fphys.2019.01140)
Supplement: Supplementary file 6 [file Table_3.pdf]

Supplementary Table 3： Metabolites in Skeletal Muscle

BIOCHEMICAL	SUPER PATHWAY
1,2-dipalmitoylglycerol	Lipid
1,5-anhydroglucitol (1,5-AG)	Carbohydrate
1,6-anhydroglucose	Xenobiotics
10-heptadecenoate (17:1n7)	Lipid
10-nonadecenoate (19:1n9)	Lipid
12,13-DiHOME	Lipid
13-HODE + 9-HODE	Lipid
1-arachidonoylglycerophosphocholine (20:4n6)*	Lipid
1-arachidonoylglycerophosphoethanolamine*	Lipid
1-arachidonoylglycerophosphoinositol*	Lipid
1-docosahexaenoylglycerol (1-monodocosahexaenoin)	Lipid
1-docosahexaenoylglycerophosphocholine (22:6n3)*	Lipid
1-docosahexaenoylglycerophosphoethanolamine*	Lipid
1-docosapentaenoylglycerophosphocholine (22:5n3)*	Lipid
1-eicosatrienoylglycerophosphocholine (20:3)*	Lipid
1-linoleoylglycerol (1-monolinolein)	Lipid
1-linoleoylglycerophosphocholine (18:2n6)	Lipid
1-linoleoylglycerophosphoethanolamine*	Lipid
1-myristoylglycerol (1-monomyristin)	Lipid
1-myristoylglycerophosphocholine (14:0)	Lipid
1-oleoylglycerol (1-monoolein)	Lipid
1-oleoylglycerophosphocholine (18:1)	Lipid
1-oleoylglycerophosphoethanolamine	Lipid
1-palmitoleoylglycerophosphocholine (16:1)*	Lipid
1-palmitoylglycerol (1-monopalmitin)	Lipid
1-palmitoylglycerophosphocholine (16:0)	Lipid
1-palmitoylglycerophosphoethanolamine	Lipid
1-palmitoylglycerophosphoglycerol*	Lipid
1-palmitoylglycerophosphoinositol*	Lipid
1-palmitoylplasmenylethanolamine*	Lipid
1-stearoylglycerol (1-monostearin)	Lipid
1-stearoylglycerophosphocholine (18:0)	Lipid
1-stearoylglycerophosphoethanolamine	Lipid
1-stearoylglycerophosphoglycerol	Lipid
1-stearoylglycerophosphoinositol	Lipid
1-stearoylglycerophosphoserine*	Lipid
1-stearoylplasmenylethanolamine*	Lipid
2-aminoadipate	Amino Acid
2-aminobutyrate	Amino Acid
2-aminoheptanoate	Lipid
2-aminooctanoate	Lipid
2-arachidonoylglycerophosphocholine*	Lipid
2-arachidonoylglycerophosphoethanolamine*	Lipid
2-arachidonoylglycerophosphoinositol*	Lipid
2-docosahexaenoylglycerophosphocholine*	Lipid
2-docosahexaenoylglycerophosphoethanolamine*	Lipid
2-docosapentaenoylglycerophosphocholine (22:5n3)*	Lipid
2-docosapentaenoylglycerophosphoethanolamine*	Lipid
2-hydroxybutyrate (AHB)	Amino Acid
2-hydroxyglutarate	Lipid
2-hydroxypalmitate	Lipid
2-hydroxystearate	Lipid
2-linoleoylglycerol (2-monolinolein)	Lipid
2-linoleoylglycerophosphocholine*	Lipid
2-linoleoylglycerophosphoethanolamine*	Lipid

2-methylbutyrylcarnitine (C5)	Amino Acid
2-oleoylglycerol (2-monoolein)	Lipid
2-oleoylglycerophosphocholine*	Lipid
2-oleoylglycerophosphoethanolamine*	Lipid
2-palmitoleoylglycerophosphocholine*	Lipid
2-palmitoylglycerol (2-monopalmitin)	Lipid
2-palmitoylglycerophosphocholine*	Lipid
2-palmitoylglycerophosphoethanolamine*	Lipid
2-phosphoglycerate	Carbohydrate
2-stearoylglycerol (2-monostearin)	Lipid
2-stearoylglycerophosphocholine*	Lipid
3-(4-hydroxyphenyl)lactate	Amino Acid
3-[3-(sulfooxy)phenyl]propanoic acid	Amino Acid
3-dehydrocarnitine	Lipid
3-hydroxybutyrate (BHBA)	Lipid
3-hydroxypyridine	Xenobiotics
3-indoxyl sulfate	Amino Acid
3-methyl-2-oxovalerate	Amino Acid
3-phosphoglycerate	Carbohydrate
4-ethylphenylsulfate	Xenobiotics
4-guanidinobutanoate	Amino Acid
4-hydroxybutyrate (GHB)	Lipid
4-hydroxyhippurate	Xenobiotics
4-methyl-2-oxopentanoate	Amino Acid
5-methylthioadenosine (MTA)	Amino Acid
5-oxoproline	Amino Acid
7-alpha-hydroxycholesterol	Lipid
7-beta-hydroxycholesterol	Lipid
9,10-DiHOME	Lipid
acetylcarnitine	Lipid
adenine	Nucleotide
adenosine	Nucleotide
adenosine 5'-diphosphate (ADP)	Nucleotide
adenosine 5'diphosphoribose	Cofactors and Vitamins
adenosine 5'-monophosphate (AMP)	Nucleotide
adrenate (22:4n6)	Lipid
alanine	Amino Acid
alanylleucine	Peptide
allantoin	Nucleotide
allo-isoleucine	Amino Acid
allo-threonine	Amino Acid
alpha-hydroxyisovalerate	Amino Acid
alpha-tocopherol	Cofactors and Vitamins
anserine	Peptide
arabinose	Carbohydrate
arabitol	Carbohydrate
arabonate	Cofactors and Vitamins
arachidate (20:0)	Lipid
arachidonate (20:4n6)	Lipid
arginine	Amino Acid
ascorbate (Vitamin C)	Cofactors and Vitamins
aspartate	Amino Acid
aspartylleucine	Peptide
azelate (nonanedioate)	Lipid
beta-alanine	Nucleotide
beta-hydroxypyruvate	Amino Acid
betaine	Amino Acid
beta-muricholate	Lipid

beta-sitosterol	Lipid
butyrylcarnitine	Lipid
campesterol	Lipid
caprate (10:0)	Lipid
caproate (6:0)	Lipid
carnitine	Lipid
carnosine	Peptide
catechol sulfate	Xenobiotics
C-glycosyltryptophan*	Amino Acid
chiro-inositol	Lipid
cholestanol	Lipid
cholesterol	Lipid
choline	Lipid
choline phosphate	Lipid
cinnamoylglycine	Xenobiotics
cis-vaccenate (18:1n7)	Lipid
citrate	Energy
citrulline	Amino Acid
creatine	Amino Acid
creatinine	Amino Acid
cysteine-glutathione disulfide	Amino Acid
cytidine	Nucleotide
cytidine 5'-diphosphocholine	Lipid
cytidine 5'-monophosphate (5'-CMP)	Nucleotide
cytidine-3'-monophosphate (3'-CMP)	Nucleotide
decanoylcarnitine	Lipid
dehydroascorbate	Cofactors and Vitamins
deoxycarnitine	Lipid
dihomo-linoleate (20:2n6)	Lipid
dihomo-linolenate (20:3n3 or n6)	Lipid
docosadienoate (22:2n6)	Lipid
docosahexaenoate (DHA; 22:6n3)	Lipid
docosapentaenoate (n3 DPA; 22:5n3)	Lipid
docosapentaenoate (n6 DPA; 22:5n6)	Lipid
eicosapentaenoate (EPA; 20:5n3)	Lipid
eicosenoate (20:1n9 or 11)	Lipid
equol sulfate	Xenobiotics
ergothioneine	Xenobiotics
erucate (22:1n9)	Lipid
erythritol	Xenobiotics
erythronate*	Carbohydrate
erythrose-4-phosphate	Carbohydrate
ethanolamine	Lipid
flavin adenine dinucleotide (FAD)	Cofactors and Vitamins
fructose	Carbohydrate
fructose 1-phosphate	Carbohydrate
fructose-6-phosphate	Carbohydrate
fucose	Carbohydrate
fumarate	Energy
galactitol (dulcitol)	Carbohydrate
gamma-aminobutyrate (GABA)	Amino Acid
gamma-glutamylleucine	Peptide
glucose	Carbohydrate
glucose 1-phosphate	Carbohydrate
glucose-6-phosphate (G6P)	Carbohydrate
glutamate	Amino Acid
glutamine	Amino Acid
glutathione, oxidized (GSSG)	Amino Acid

glutathione, reduced (GSH)	Amino Acid
glycerate	Carbohydrate
glycerol	Lipid
glycerol 2-phosphate	Xenobiotics
glycerol 3-phosphate (G3P)	Lipid
glycerophosphorylcholine (GPC)	Lipid
glycine	Amino Acid
glycylleucine	Peptide
glycylvaline	Peptide
guanosine	Nucleotide
guanosine 5'- monophosphate (5'-GMP)	Nucleotide
hexanoylcarnitine	Lipid
hippurate	Xenobiotics
histamine	Amino Acid
histidine	Amino Acid
homoserine	Amino Acid
homostachydrine*	Xenobiotics
hydroxybutyrylcarnitine*	Lipid
hypotaurine	Amino Acid
hypoxanthine	Nucleotide
imidazole propionate	Amino Acid
indolelactate	Amino Acid
inosine	Nucleotide
inosine 5'-monophosphate (IMP)	Nucleotide
inositol 1-phosphate (I1P)	Lipid
Isobar: fructose 1,6-diphosphate, glucose 1,6-diphosphate, my	Carbohydrate
isobutyrylcarnitine	Amino Acid
isoleucine	Amino Acid
isoleucylglycine	Peptide
isovalerylcarnitine	Amino Acid
isovalerylglycine	Amino Acid
lactate	Carbohydrate
leucine	Amino Acid
leucylglycine	Peptide
linoleate (18:2n6)	Lipid
linolenate [alpha or gamma; (18:3n3 or 6)]	Lipid
lysine	Amino Acid
malate	Energy
malonylcarnitine	Lipid
maltose	Carbohydrate
maltotetraose	Carbohydrate
maltotriose	Carbohydrate
mannitol	Carbohydrate
mannose	Carbohydrate
mannose-6-phosphate	Carbohydrate
margarate (17:0)	Lipid
mead acid (20:3n9)	Lipid
methionine	Amino Acid
methionine sulfoxide	Amino Acid
methyl-beta-glucopyranoside	Carbohydrate
methylphosphate	Nucleotide
myo-inositol	Lipid
myristate (14:0)	Lipid
myristoleate (14:1n5)	Lipid
myristoylcarnitine	Lipid
N-acetylaspartate (NAA)	Amino Acid
N-acetyl-aspartyl-glutamate (NAAG)	Amino Acid
N-acetylcarnosine	Peptide

N-acetylmethionine	Amino Acid
N-acetylthreonine	Amino Acid
nicotinamide	Cofactors and Vitamins
nicotinamide adenine dinucleotide (NAD+)	Cofactors and Vitamins
nicotinate	Cofactors and Vitamins
nonadecanoate (19:0)	Lipid
octanoylcarnitine	Lipid
oleate (18:1n9)	Lipid
oleic ethanolamide	Lipid
oleoylcarnitine	Lipid
ophthalmate	Amino Acid
ornithine	Amino Acid
palmitate (16:0)	Lipid
palmitoleate (16:1n7)	Lipid
palmitoyl ethanolamide	Lipid
palmitoyl sphingomyelin	Lipid
palmitoylcarnitine	Lipid
pantothenate	Cofactors and Vitamins
p-cresol sulfate	Amino Acid
pentadecanoate (15:0)	Lipid
phenol sulfate	Amino Acid
phenylacetylglycine	Amino Acid
phenylalanine	Amino Acid
phenylalanylglutamate	Peptide
phenylalanylserine	Peptide
phosphate	Energy
phosphoenolpyruvate (PEP)	Carbohydrate
phosphoethanolamine	Lipid
phosphopantetheine	Cofactors and Vitamins
pipecolate	Amino Acid
proline	Amino Acid
propionylcarnitine	Lipid
pseudouridine	Nucleotide
putrescine	Amino Acid
pyroglutamine*	Amino Acid
pyrophosphate (PPi)	Energy
pyruvate	Carbohydrate
ribitol	Carbohydrate
ribose	Carbohydrate
ribose 5-phosphate	Carbohydrate
ribulose	Carbohydrate
ribulose/xylulose 5-phosphate	Carbohydrate
S-adenosylhomocysteine (SAH)	Amino Acid
sarcosine (N-Methylglycine)	Amino Acid
scyllo-inositol	Lipid
sedoheptulose-7-phosphate	Carbohydrate
serine	Amino Acid
serylisoleucine*	Peptide
serylleucine	Peptide
serylphenyalanine	Peptide
S-lactoylglutathione	Amino Acid
S-methylcysteine	Amino Acid
sorbitol	Carbohydrate
spermidine	Amino Acid
sphingosine	Lipid
squalene	Lipid
stachydrine	Xenobiotics
stearate (18:0)	Lipid

stearidonate (18:4n3)	Lipid
stearoyl sphingomyelin	Lipid
stearoylcarnitine	Lipid
succinylcarnitine	Energy
taurine	Amino Acid
tauro(alpha + beta)muricholate	Lipid
taurochenodeoxycholate	Lipid
taurocholate	Lipid
taurodeoxycholate	Lipid
tauroursodeoxycholate	Lipid
threonine	Amino Acid
trans-4-hydroxyproline	Amino Acid
trans-urocanate	Amino Acid
tryptophan	Amino Acid
tyrosine	Amino Acid
uracil	Nucleotide
urate	Nucleotide
urea	Amino Acid
uridine	Nucleotide
uridine monophosphate (5' or 3')	Nucleotide
valerate	Lipid
valerylcarnitine	Lipid
valine	Amino Acid
valylglutamine	Peptide
valylleucine	Peptide
xanthine	Nucleotide
xylitol	Carbohydrate
xylonate	Carbohydrate
xylose	Carbohydrate
xylulose	Carbohydrate
