# Supplementary material for: BCAA Catabolic Defect Alters Glucose Metabolism in Lean Mice
Source: Front Physiol. 2019 Sep 4;10:1140. doi: 10.3389/fphys.2019.01140 (PMC6738029; doi:10.3389/fphys.2019.01140)
Supplement: Supplementary file 7 [file Table_4.pdf]

Supplementary Table 4：Metabolites in Adipose Tissue

BIOCHEMICAL	SUPER_PATHWAY
1,5-anhydroglucitol (1,5-AG)	Carbohydrate
1-arachidonoylglycerophosphoethanolamine*	Lipid
1-arachidonoylglycerophosphoinositol*	Lipid
1-linoleoylglycerophosphocholine (18:2n6)	Lipid
1-linoleoylglycerophosphoethanolamine*	Lipid
1-oleoylglycerophosphocholine (18:1)	Lipid
1-oleoylglycerophosphoethanolamine	Lipid
1-oleoylglycerophosphoinositol*	Lipid
1-oleoylplasmenylethanolamine*	Lipid
1-palmitoylglycerol (1-monopalmitin)	Lipid
1-palmitoylglycerophosphocholine (16:0)	Lipid
1-palmitoylglycerophosphoethanolamine	Lipid
1-palmitoylplasmenylethanolamine*	Lipid
1-stearoylglycerophosphocholine (18:0)	Lipid
1-stearoylglycerophosphoethanolamine	Lipid
1-stearoylglycerophosphoinositol	Lipid
1-stearoylglycerophosphoserine*	Lipid
1-stearoylplasmenylethanolamine*	Lipid
10-heptadecenoate (17:1n7)	Lipid
10-nonadecenoate (19:1n9)	Lipid
12,13-DiHOME	Lipid
12-HETE	Lipid
13-HODE + 9-HODE	Lipid
13-methylmyristic acid	Lipid
15-methylpalmitate (isobar with 2-methylpalmitate)	Lipid
17-methylstearate	Lipid
2-aminoadipate	Amino Acid
2-aminobutyrate	Amino Acid
2-aminoheptanoate	Lipid
2-hydroxyglutarate	Lipid
2-methylbutyrylcarnitine (C5)	Amino Acid
3-hydroxybutyrate (BHBA)	Lipid
5-dodecenoate (12:1n7)	Lipid
5-oxoproline	Amino Acid
acetylcarnitine	Lipid
adenine	Nucleotide
adenosine	Nucleotide
adenosine 5'-diphosphoribose (ADP-ribose)	Cofactors and Vitamins
adenosine 5'-monophosphate (AMP)	Nucleotide
adrenate (22:4n6)	Lipid
alanine	Amino Acid
alpha-tocopherol	Cofactors and Vitamins
arachidate (20:0)	Lipid
arachidonate (20:4n6)	Lipid
arginine	Amino Acid
ascorbate (Vitamin C)	Cofactors and Vitamins
asparagine	Amino Acid
aspartate	Amino Acid
beta-alanine	Nucleotide
beta-hydroxyisovaleroylcarnitine	Amino Acid
beta-muricholate	Lipid
betaine	Amino Acid
butyrylcarnitine	Lipid
carnitine	Lipid
catechol sulfate	Xenobiotics

cholate	Lipid
cholestanol	Lipid
cholesterol	Lipid
choline phosphate	Lipid
cis-aconitate	Energy
cis-vaccenate (18:1n7)	Lipid
citrate	Energy
citrulline	Amino Acid
corticosterone	Lipid
creatine	Amino Acid
cysteine	Amino Acid
cysteine-glutathione disulfide	Amino Acid
cytidine	Nucleotide
cytidine 5'-monophosphate (5'-CMP)	Nucleotide
dihomo-linoleate (20:2n6)	Lipid
dihomo-linolenate (20:3n3 or n6)	Lipid
docosahexaenoate (DHA; 22:6n3)	Lipid
docosapentaenoate (n3 DPA; 22:5n3)	Lipid
docosapentaenoate (n6 DPA; 22:5n6)	Lipid
eicosapentaenoate (EPA; 20:5n3)	Lipid
eicosenoate (20:1n9 or 11)	Lipid
ergothioneine	Xenobiotics
erucate (22:1n9)	Lipid
erythritol	Xenobiotics
ethanolamine	Lipid
flavin adenine dinucleotide (FAD)	Cofactors and Vitamins
fructose	Carbohydrate
fructose-6-phosphate	Carbohydrate
gamma-aminobutyrate (GABA)	Amino Acid
glucose	Carbohydrate
glucose-6-phosphate (G6P)	Carbohydrate
glutamate	Amino Acid
glutamine	Amino Acid
glutathione, oxidized (GSSG)	Amino Acid
glycerate	Carbohydrate
glycerol	Lipid
glycerol 3-phosphate (G3P)	Lipid
glycerophosphoethanolamine	Lipid
glycerophosphorylcholine (GPC)	Lipid
glycine	Amino Acid
guanosine	Nucleotide
guanosine 5'- monophosphate (5'-GMP)	Nucleotide
heme	Cofactors and Vitamins
hydroxybutyrylcarnitine*	Lipid
hypotaurine	Amino Acid
hypoxanthine	Nucleotide
inosine	Nucleotide
inosine 5'-monophosphate (IMP)	Nucleotide
inositol 1-phosphate (I1P)	Lipid
Isobar: fructose 1,6-diphosphate, glucose 1,6-diphosphate, my	Carbohydrate
isobutyrylcarnitine	Amino Acid
isoleucine	Amino Acid
isovalerylcarnitine	Amino Acid
leucine	Amino Acid
linoleate (18:2n6)	Lipid
linolenate [alpha or gamma; (18:3n3 or 6)]	Lipid
lysine	Amino Acid
malate	Energy

maltose	Carbohydrate
mannose-6-phosphate	Carbohydrate
margarate (17:0)	Lipid
mead acid (20:3n9)	Lipid
methionine	Amino Acid
myo-inositol	Lipid
myristate (14:0)	Lipid
myristoleate (14:1n5)	Lipid
N-acetylaspartate (NAA)	Amino Acid
N-acetylglutamate	Amino Acid
N-oleoyltaurine	Lipid
N-palmitoyltaurine	Lipid
N-stearoyltaurine	Lipid
nicotinamide	Cofactors and Vitamins
nicotinate	Cofactors and Vitamins
nonadecanoate (19:0)	Lipid
oleate (18:1n9)	Lipid
oleic ethanolamide	Lipid
ornithine	Amino Acid
p-cresol sulfate	Amino Acid
palmitate (16:0)	Lipid
palmitoleate (16:1n7)	Lipid
palmitoyl ethanolamide	Lipid
palmitoyl sphingomyelin	Lipid
pantothenate	Cofactors and Vitamins
pentadecanoate (15:0)	Lipid
phenol sulfate	Amino Acid
phenylalanine	Amino Acid
phosphate	Energy
phosphoethanolamine	Lipid
proline	Amino Acid
propionylcarnitine	Lipid
prostaglandin A2	Lipid
prostaglandin B2	Lipid
prostaglandin E1	Lipid
prostaglandin E2	Lipid
prostaglandin F2alpha	Lipid
putrescine	Amino Acid
S-adenosylhomocysteine (SAH)	Amino Acid
scyllo-inositol	Lipid
serine	Amino Acid
spermidine	Amino Acid
spermine	Amino Acid
stachydrine	Xenobiotics
stearate (18:0)	Lipid
stearidonate (18:4n3)	Lipid
stearoyl sphingomyelin	Lipid
taurine	Amino Acid
tauro(alpha + beta)muricholate	Lipid
taurochenodeoxycholate	Lipid
taurocholate	Lipid
taurodeoxycholate	Lipid
tauroursodeoxycholate	Lipid
threonate	Cofactors and Vitamins
threonine	Amino Acid
trans-4-hydroxyproline	Amino Acid
tryptophan	Amino Acid
tyrosine	Amino Acid

UDP-acetylglucosamine/galactosamine	Carbohydrate
UDP-glucose (isobar with UDP-galactose)	Carbohydrate
uracil	Nucleotide
urate	Nucleotide
urea	Amino Acid
uridine	Nucleotide
uridine monophosphate (5' or 3')	Nucleotide
valine	Amino Acid
xanthine	Nucleotide
